# Cold Shock Proteins Promote Nisin Tolerance in *Listeria monocytogenes* Through Modulation of Cell Envelope Modification Responses

**DOI:** 10.3389/fmicb.2021.811939

**Published:** 2021-12-24

**Authors:** Francis Muchaamba, Joseph Wambui, Roger Stephan, Taurai Tasara

**Affiliations:** Institute for Food Safety and Hygiene, Vetsuisse Faculty University of Zürich, Zurich, Switzerland

**Keywords:** *Listeria monocytogenes*, cold shock protein, nisin, tolerance, cell envelope

## Abstract

*Listeria monocytogenes* continues to be a food safety challenge owing to its stress tolerance and virulence traits. Several listeriosis outbreaks have been linked to the consumption of contaminated ready-to-eat food products. Numerous interventions, including nisin application, are presently employed to mitigate against *L. monocytogenes* risk in food products. In response, *L. monocytogenes* deploys several defense mechanisms, reducing nisin efficacy, that are not yet fully understood. Cold shock proteins (Csps) are small, highly conserved nucleic acid-binding proteins involved in several gene regulatory processes to mediate various stress responses in bacteria. *L. monocytogenes* possesses three *csp* gene paralogs; *cspA*, *cspB*, and *cspD*. Using a panel of single, double, and triple *csp* gene deletion mutants, the role of Csps in *L. monocytogenes* nisin tolerance was examined, demonstrating their importance in nisin stress responses of this bacterium. Without *csp* genes, a *L. monocytogenes* Δ*cspABD* mutant displayed severely compromised growth under nisin stress. Characterizing single (Δ*cspA*, Δ*cspB*, and Δ*cspD*) and double (Δ*cspBD*, Δ*cspAD*, and Δ*cspAB*) *csp* gene deletion mutants revealed a hierarchy (*cspD* > *cspB* > *cspA*) of importance in *csp* gene contributions toward the *L. monocytogenes* nisin tolerance phenotype. Individual eliminations of either *cspA* or *cspB* improved the nisin stress tolerance phenotype, suggesting that their expression has a curbing effect on the expression of nisin resistance functions through CspD. Gene expression analysis revealed that Csp deficiency altered the expression of DltA, MprF, and penicillin-binding protein-encoding genes. Furthermore, the Δ*cspABD* mutation induced an overall more electronegative cell surface, enhancing sensitivity to nisin and other cationic antimicrobials as well as the quaternary ammonium compound disinfectant benzalkonium chloride. These observations demonstrate that the molecular functions of Csps regulate systems important for enabling the constitution and maintenance of an optimal composed cell envelope that protects against cell-envelope-targeting stressors, including nisin. Overall, our data show an important contribution of Csps for *L. monocytogenes* stress protection in food environments where antimicrobial peptides are used. Such knowledge can be harnessed in the development of better *L. monocytogenes* control strategies. Furthermore, the potential that Csps have in inducing cross-protection must be considered when combining hurdle techniques or using them in a series.

## Introduction

*Listeria monocytogenes* is a serious public health and food safety challenge and a major economic burden worldwide. Listeriosis, which is caused by this bacterium, is a serious foodborne disease responsible for severe clinical illness with high rates of hospitalization and mortality among those with diminished immunity as well as abortions and stillbirths in pregnant women [European Food Safety Authority [EFSA], 2017; Centers for Disease Control [CDC], 2018]. Low temperatures, elevated salt levels, low water activity, acidity, and bacteriocins are food-associated environmental conditions constituting the stress situations facing *L. monocytogenes* along the food supply chain (Gandhi and Chikindas, 2007; Burgess et al., 2016; Bucur et al., 2018; Wiktorczyk-Kapischke et al., 2021). In response, this bacterium is endowed with different physiological and molecular stress response mechanisms for adaptation and resistance to such food-related harsh environmental conditions (Bucur et al., 2018; Wiktorczyk-Kapischke et al., 2021).

Nisin is a commonly used bacteriocin that mitigates against spoilage and pathogenic foodborne bacteria, including *L. monocytogenes* (Cotter et al., 2013; Zhou et al., 2014; Alvarez-Sieiro et al., 2016). Nisin inactivates bacteria through a dual mechanism targeting cell membrane and cell wall synthesis (Bruno et al., 1992; Abee et al., 1994; Wiedemann et al., 2001; Alvarez-Sieiro et al., 2016). The efficacy of nisin against *L. monocytogenes* is, however, hampered as this bacterium possesses various molecular and physiological defense mechanisms that confer intrinsic nisin-resistant responses, including cell wall- and membrane-associated changes (Gravesen et al., 2002; NicAogáin and O’Byrne, 2016; Bucur et al., 2018). Some of the nisin stress mitigation responses documented in this bacterium to date include cell envelope composition and net cell surface charge changes that are mediated through D-alanylation and lysinylation of cell wall teichoic acids and membrane phospholipids, respectively (Abachin et al., 2002; Thedieck et al., 2006). These responses involving the Dlt and MprF protein systems, respectively, and are in part regulated through the VirABRS four-component regulatory protein system (Collins et al., 2010a; Grubaugh et al., 2018; Jiang et al., 2019). In addition, other nisin-protective response molecular mechanisms documented in this bacterium are orchestrated through elaborate regulatory cascade loops that involve CesRK, LisRK, and LiaFSR regulatory protein systems, which, upon sensing nisin stress, consequently implement protection responses through expression regulation of various genes in their regulons (Cotter et al., 2002; Kallipolitis et al., 2003; Fritsch et al., 2011; Nielsen et al., 2012; Bergholz et al., 2013: Draper et al., 2015).

Other proteins, such as AnrAB, TelA, and GadD1, as well as the alternative sigma factors, SigB and SigL, have been found to contribute toward nisin stress tolerance of this bacterium, but the precise mechanisms of their involvement remain to be fully elucidated (Begley et al., 2006, 2010; Palmer et al., 2009; Collins et al., 2010a,b; Stincone et al., 2020). Wall teichoic acid (WTA) decoration with L-rhamnose, which is mediated by RmlT (rhamnosyltransferase) and RmlABCD proteins, has also been postulated to increase tolerance to antimicrobial peptides, such as nisin, by acting as a barrier that delays nisin passage through the cell wall, hence limiting access to and/or interactions with the cell membrane (Carvalho et al., 2015). Furthermore, it has been suggested that *L. monocytogenes* enforces other cell wall structural changes that prevent nisin from accessing the cell membrane, thereby increasing resilience against it (Kaur et al., 2012).

Cold shock proteins (Csps) are nucleic acid binding proteins that serve as global gene expression regulators involved in different cellular and physiological processes to facilitate bacterial growth under different conditions, including stress adaptation and virulence responses (Keto-Timonen et al., 2016; Muchaamba et al., 2021). Csp modulation of global gene expression regulation events is mediated through nucleic acid binding and inhibitory secondary structure melting events that modulate transcription, translation, and mRNA stability processes (Feng et al., 2001; Phadtare et al., 2002; Schärer et al., 2013; Hudson and Ortlund, 2014; Michaux et al., 2017; Caballero et al., 2018). Csp functions have been found to protect against a broad range of stress conditions in bacteria, including low temperatures, nutrient deprivation, high osmolarity, low pH, antibiotics, and oxidative stress (Phadtare and Severinov, 2010; Keto-Timonen et al., 2016; Michaux et al., 2017; Muchaamba et al., 2021). Furthermore, phenotypes such as virulence, extracellular motility, cell aggregation, and biofilm production have all been found to be Csp dependent in different bacteria (Michaux et al., 2012, 2017; Wang et al., 2014)—for instance, in *Salmonella enterica* and *Staphylococcus aureus*, Csps are important for biofilm production (Sahukhal and Elasri, 2014; Michaux et al., 2017), while in *Escherichia coli*, *csp* genes were induced upon exposure to antibiotics (Cruz-Loya et al., 2019), whereas in *Bacillus subtilis*, the deletion of all its *csp* genes resulted in a lethal phenotype (Graumann et al., 1997).

The precise molecular mechanisms and events through which Csps functionally contribute to such a broad range of cellular processes and phenotypes remain to be fully elucidated. The deletion of *csp* genes in *E. coli*, *S. enterica*, *B. subtilis*, and *Brucella melitensis* among other bacteria has shown that these proteins contribute to global gene expression regulation, as their loss affected the expression of genes associated with different physiological processes and bacterial phenotypes (Willimsky et al., 1992; Graumann et al., 1997; Michaux et al., 2012; Wang et al., 2014, 2016; Caballero et al., 2018; Cruz-Loya et al., 2019)—for example, in *B. melitensis* and *S. aureus*, *cspA* removal altered the expression of various genes involved in metabolism (Wang et al., 2016; Caballero et al., 2018).

*Listeria monocytogenes* possesses three Csp paralogs (CspA/L, CspB, and CspD). Previous studies have linked Csps to cold, osmotic, oxidative, and desiccation stress tolerance responses as well as virulence, cell aggregation, biofilm production, and motility in this bacterium (Schmid et al., 2009; Loepfe et al., 2010; Schärer et al., 2013; Eshwar et al., 2017; Kragh et al., 2020). Redundancy and division of labor have also been noted among these three Csps regarding their functional contributions to the different *L. monocytogenes* phenotypes (Muchaamba et al., 2021). CspA is most relevant in cold and desiccation tolerance, whereas CspB is most important in virulence responses. CspD, on the other hand, seems to be an “all-weather” Csp with important functional contributions to both virulence and stress response phenotypes, but neither the regulatory mechanisms behind this Csp division of labor nor the mechanistic pathways of Csp involvement in stress protection and virulence responses in this bacterium are yet known. The loss of Csps is linked to a diminished expression of key virulence genes, including *prfA* and *hly*, thus showing that Csp regulatory inputs contribute to virulence expression regulation in this bacterium (Schärer et al., 2013; Eshwar et al., 2017). Meanwhile, recently, there were possible links suggested between Csps and nisin stress protection responses in this bacterium since *csp* mRNAs were detected among transcripts regulated in response to nisin exposure of *L. monocytogenes* (Liu et al., 2013; Wu et al., 2018). We thus hypothesized that Csps might be functionally important for the intrinsic nisin protection responses in *L. monocytogenes*. In the present study, we therefore examined the functional contribution of Csps to nisin tolerance in *L. monocytogenes*.

## Materials and Methods

### Bacterial Strains and Growth Conditions

*L. monocytogenes* EGDe wild type (WT) and *csp* (Δ*cspA*, Δ*cspB*, Δ*cspD*, Δ*cspBD*, Δ*cspAD*, Δ*cspAB*, and Δ*cspABD*) deletion mutant strains that have been previously described were used (Schmid et al., 2009; Table 1). A selection of genetic complementation strains was created and used for the phenotypic validation of our observations (Table 1). All strains were preserved at −80°C in brain heart infusion (BHI; Oxoid Ltd., Hampshire, United Kingdom) broth plus 20% glycerol (Sigma-Aldrich Co., Missouri, United States) and resuscitated by plating out on BHI agar and incubating for 24–36 h at 37°C. Single colonies from each strain were inoculated in BHI broth (5 ml) and cultivated aerobically for 16 h at 37°C and 150 rpm. The primary cultures generated were subcultured (1:100) in BHI and cultivated to give secondary-stationary-phase-stage cultures that were used in the experiments unless otherwise mentioned.

**TABLE 1:** Strains used in this study.

Strain ID	Description	References
EGDe	Reference strain, LII, serotype 1/2a, CC9	Glaser et al., 2001
**Δ*csp* strains**		
EGDe_Δ*cspA*	In-frame *cspA* deletion	Schmid et al., 2009
EGDe_Δ*cspB*	In-frame *cspB* deletion	Schmid et al., 2009
EGDe_Δ*cspD*	In-frame *cspD* deletion	Schmid et al., 2009
EGDe_Δ*cspAB*	In-frame *cspA* and *B* deletions	Schmid et al., 2009
EGDe_Δ*cspAD*	In-frame *cspA* and *D* deletions	Schmid et al., 2009
EGDe_Δ*cspBD*	In-frame *cspB* and *D* deletions	Schmid et al., 2009
*EGDe_ΔcspABD*	In-frame *cspA, B*, and *D* deletions	Schmid et al., 2009
EGDe_Δ*cspA*::pPL2- *cspA*	EGDe *cspA* deletion complemented with pPL2-*cspA*	This study
EGDe_Δ*cspD*::pPL2-*cspD*	In-frame *cspD* deletion complemented with pPL2-*cspA*	This study
*EGDe_ΔcspABD*::pPL2-*cspA*	In-frame *cspA*, *B*, and *D* deletions complemented with pPL2-*cspA*	This study
*EGDe_ΔcspABD*::pPL2-*cspB*	In-frame *cspA*, *B*, and *D* deletions complemented with pPL2-*cspB*	This study
*EGDe_ΔcspABD*::pPL2-*cspD*	In-frame *cspA*, *B*, and *D* deletions complemented with pPL2-*cspD*	This study
**Plasmids**		
pPL2	Plasmid vector	Lauer et al., 2002
pPL2-*cspA*	pPL2 with *cspA* sequence and 5′ flanking region	This study
pPL2-*cspB*	pPL2 with *cspB* sequence and 5′ flanking region	This study
pPL2-*cspD*	pPL2 with *cspD* sequence and 5′ flanking region	This study

*L, lineage; CC, clonal complex.*

### Complementation of *csp* Gene Deletion Mutant Strains

Genetic complementation of the *csp* deletion mutants was performed as previously described (Schmid et al., 2009). Individual *csp* genes, including their upstream sequences and native promoter regions in *L. monocytogenes* EGDe genomic DNA, were PCR-amplified and seamlessly cloned into the pPL2 integrative plasmid vector (Lauer et al., 2002) using In-Fusion Cloning System (Takara Bio SAS Europe, Saint-Germain-en-Laye, France). The *csp* gene complementation pPL2 plasmids generated were purified, introduced into *csp* deletion mutants by electroporation, and chromosomally integrated as previously described (Schmid et al., 2009). All the plasmid constructs and gene complementation mutants were confirmed through DNA sequencing.

### Growth Evaluation Under Nisin Stress

Growth under nisin stress was determined using microtiter plate-based broth assays. Secondary cultures prepared as detailed above and diluted to 10^5^ colony-forming units (CFU)/ml in BHI were distributed (100 μl) into a 96-well microtiter plate (non-tissue-culture-treated; Corning Incorporated, New York, United States) in duplicates, after which normal (control, 100 μl) or nisin-supplemented (10 ppm; Sigma-Aldrich Co., Missouri, United States) BHI broth was added, achieving final nisin working concentrations of 0 (control) and 5 ppm (BHI nisin). The plates were incubated at 37°C with continuous medium shaking, and growth was monitored through optical density measurements determined at 600 nm (OD_600_; Synergy HT OD reader; BioTek, Lucerne, Switzerland) over 24 h. To evaluate growth under dual nisin and cold stress, strains (10^7^ CFU/ml) were grown at 8°C in 10-ml-BHI tubes supplemented with 0 and 5 ppm nisin. The tubes were incubated at 8°C without shaking, and growth was monitored through OD_600_ measurements every 24 h for the first 7 days and then every 48 h thereafter for 19 days using a Synergy HT OD reader. The growth parameters [lag phase duration (LPD), maximum growth rate (MGR), and area under the curve (AUC)] were determined from the growth curve data generated using the R package “*opm*” (Göker, 2016; Göker et al., 2016) and GraphPad Prism [version 9.2.0 (283), GraphPad Software, San Diego, CA, United States].

### Nisin Stress Survival Assays

Nisin stress survival was evaluated at 7.5 ppm. Secondary-stationary-phase *L. monocytogenes* cultures, prepared as described above, were standardized to OD_600_ 1.0 (10^9^ CFU/ml), inoculated (1:100) in BHI broth supplemented with 7.5 ppm nisin, and incubated at 37°C for 60 min. The cultures were sampled before (*t*_0_) and after 60 min of nisin stress exposure (*t*_60_) and then 10-fold serially diluted and plated out on BHI agar plates that were incubated for 36 h at 37°C, followed by viable cell count determination. The survival rates were determined as the percentage difference between colony-forming units before and after nisin exposure. Strains were assessed in three independent biological experiments performed in duplicates.

### Benzalkonium Chloride Survival Assays

Benzalkonium chloride (BC) stress survival was evaluated at 10 ppm. Secondary-stationary-phase cultures of *L. monocytogenes* EGDe strains (WT and Δ*cspABD*), prepared as outlined above, were standardized to OD_600_ 1.0 (10^9^ CFU/ml), inoculated (1:10) in phosphate-buffered saline (PBS) containing 10 ppm BC (Sigma-Aldrich, Buchs, Switzerland), and incubated at 25°C without shaking. After 0 and 15 min (*t*_0_ and *t*_15_) of incubation, the samples were diluted (1:10) in Dey Engley neutralizing broth (Sigma-Aldrich Co., Missouri, United States). The neutralized samples were 10-fold serially diluted in PBS and plated out on BHI agar plates that were then incubated for 36 h at 37°C, followed by viable cell count determination. The survival rates were determined as the percentage difference between colony-forming units after BC treatment (*t*_15_) relative to CFU counts before BC treatment (*t*_0_). To assess growth under BC stress, the strains were grown in BHI supplemented with 1.2 ppm BC at 37°C for 24 h using the same 96-well plate setup described for nisin. All experiments were conducted in three independent biological experiments performed in duplicates.

### Reverse Transcription Quantitative PCR Analysis

Reverse transcription quantitative PCR (RT-qPCR) was applied to assess the impact of nisin stress and Csp deficiency on gene expression. The targeted genes and primers that were used in this study are listed in Supplementary Table 1. To assess nisin impact on *csp* gene expression, *L. monocytogenes* EGDe WT strain cultures were diluted (1:100) in 50 ml of normal and nisin (5 ppm)-supplemented BHI in 200-ml conical flasks. The cultures were aerobically cultivated for 16 h at 37°C and 150 rpm. To assess Csp deficiency impact on the expression of selected nisin response genes, EGDe WT and Δ*cspABD* secondary cultures were similarly diluted (1:100) in normal and nisin (1.5 ppm)-supplemented BHI and cultivated to the late exponential growth phase (OD_600_ 1.0) stage. To evaluate Csp deficiency impact on *rmlT* (*lmo1085*; rhamnosyltransferase) expression, secondary-stationary-phase-stage cultures (10 ml) of EGDe WT and Δ*cspABD* strains grown in BHI, as described above, were centrifuged (6,000 rpm for 5 min). The supernatant was discarded, and the pellets were washed once in PBS, resuspended in 10 ml phenol-red minimal media [10 g pancreatic digest of casein, 5 g sodium chloride, 0.018 g phenol red per litre (Salazar et al., 2013)] containing L-rhamnose (5g/L) as the sole C-source, and then incubated for 4 h at 37°C. One milliliter of aliquot per sample was harvested in RNA Protect Bacteria reagent (Qiagen, Hombrechtikon, Switzerland) and resuspended in 0.5 ml RNeasy Plus Mini Kit lysis buffer (Qiagen, Hombrechtikon, Switzerland). RNA, isolated as previously described (Kropac et al., 2019), was quantified (Quantus Fluorometer; Promega, Wisconsin, United States) and quality-controlled (BioAnalyzer; Agilent Technologies, United States). One microgram of RNA (RNA integrity number ≥ 8.0) was converted to cDNA using the Quantitect reverse transcription kit (Qiagen GmbH, Hilden, Germany). PCR reactions were performed using the light cycler LC 480 (Roche Molecular Diagnostics, Risch-Rotkreuz, Switzerland) instrument in 20 μl. Each reaction contained 5 μl (14 ng of 1:10 dilution) cDNA, 5 μl (0.4 μM) of primers, and 10 μl of 2X LC*^R^* 480 SYBR Green I master mix (Roche Molecular Diagnostics, Penzberg, Germany). DNA contamination of RNA samples was controlled for by including no reverse transcription (no-RT) controls. The RT-qPCR cycling conditions were as previously described (Kropac et al., 2019). Relative cDNA quantification was performed using the Light Cycler 480 Relative Quantification Software (Roche Molecular Diagnostics). The mRNA amounts were normalized using 16S rRNA as a reference gene (Tasara and Stephan, 2007). The samples were analyzed in three independent biological experiments performed with two technical replicates.

### Cytochrome c Binding

To compare cell surface positive charge between *L. monocytogenes* EGDe WT and Δ*cspABD* strains, the cytochrome c binding assay was performed as previously described (Kang et al., 2015). Briefly, secondary-stationary-phase cultures of the strains diluted (1:100) and grown (37°C and 150 rpm) to the late exponential phase [OD 1.0 (10^9^ CFU/ml)] in BHI were harvested by centrifugation (8,000 × *g* for 5 min) and washed twice (8,000 × *g* for 5 min) with 20 mM MOPS [3-(N morpholino) propanesulfonic acid] buffer (pH 7) (Sigma-Aldrich Co., Missouri, United States). After washing, the cells were standardized to OD_0.25_ (10^8^ CFU/ml) in MOPS buffer, and then cytochrome c (Sigma-Aldrich, St. Louis, MO, United States) was added at a concentration of 50 μg/ml. The mixture was incubated for 15 min at room temperature. After incubation, the OD_530_ of the samples was determined (OD_530_ with cells) followed by centrifugation (13,000 × *g* for 5 min). The supernatant was collected, and its OD_530_ was measured (OD_530_ without cells). Cytochrome c binding was calculated and expressed as a percentage as follows:


$$\%\mathrm{bound}\mathrm{cytochrome}c=100\left(1-\frac{{\text{OD}}_{530}\,\mathrm{with}\,\mathrm{cells}}{{\text{OD}}_{530}\,\mathrm{without}\,\mathrm{cells}}\right)$$

### Antibiotic Sensitivity Testing

To investigate further the effects of *csp* gene removal on cell wall and cell membrane systems, we next compared the sensitivity of the WT strain and the *csp* deletion mutants to cell wall- and cell membrane-targeting antibiotics. Bacteria were grown overnight on blood agar plates at 37°C, after which 0.5 McFarland standard density bacteria solutions were prepared and spread onto Muller Hinton plus blood agar plates to cover the whole surface. Ampicillin, daptomycin, polymyxin B, and vancomycin E tests strips were then placed on the center of each plate, and sensitivity to each antibiotic was determined in accordance with the recommendations of the manufacturer (Biomerieux, Lyon, France). To simulate conditions under which nisin stress sensitivity was tested, daptomycin and vancomycin sensitivity was also determined using BHI agar plates. The results were assessed after 48 h of incubation at 37°C.

### Statistical Analyses

Statistical analysis of the data was done using GraphPad Prism (Version 9.2.0 (283), GraphPad Software, San Diego, CA, United States). One-way ANOVA with *post-hoc* Tukey honestly significant difference tests and *t*-tests were used to assess the significance of differences between the EGDe WT and the *csp* mutant strains. *P*-values < 0.05 were considered to be statistically significant.

## Results

### Loss of Csps Attenuates Nisin Resistance

**FIGURE 1:**
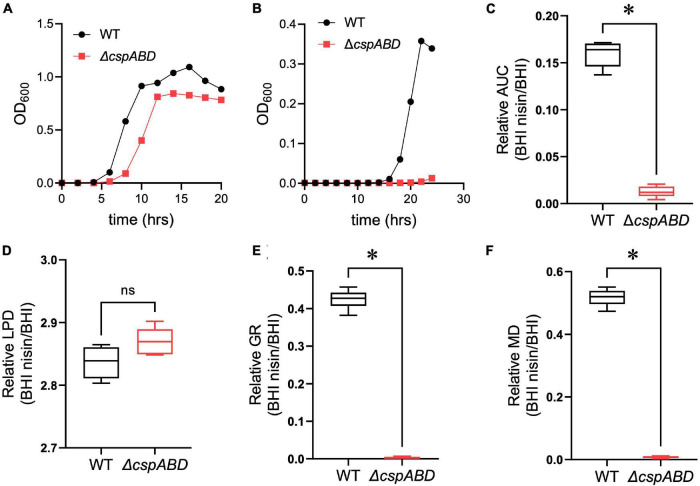
Csp deficiency severely impairs nisin stress growth efficiency in *L. monocytogenes*. **(A,B)** Optical density measurement-based growth curves of *L. monocytogenes* EGDe wild-type (WT) and Δ*cspABD* strains cultivated (37°C and 150 rpm) in **(A)** normal and **(B)** nisin (5 ppm)-supplemented BHI. **(C–F)** Box plots summarizing the relative nisin stress growth parameters (area under the curve, lag phase duration, growth rate, and maximum cell density) of EGDe WT and Δ*cspABD* strains normalized for each strain to the growth parameters of controls grown in BHI without stress. ^∗^*P* < 0.05, significant differences between the WT and Δ*cspABD* strains identified using the *t*-test for comparison of independent samples.

**FIGURE 2:**
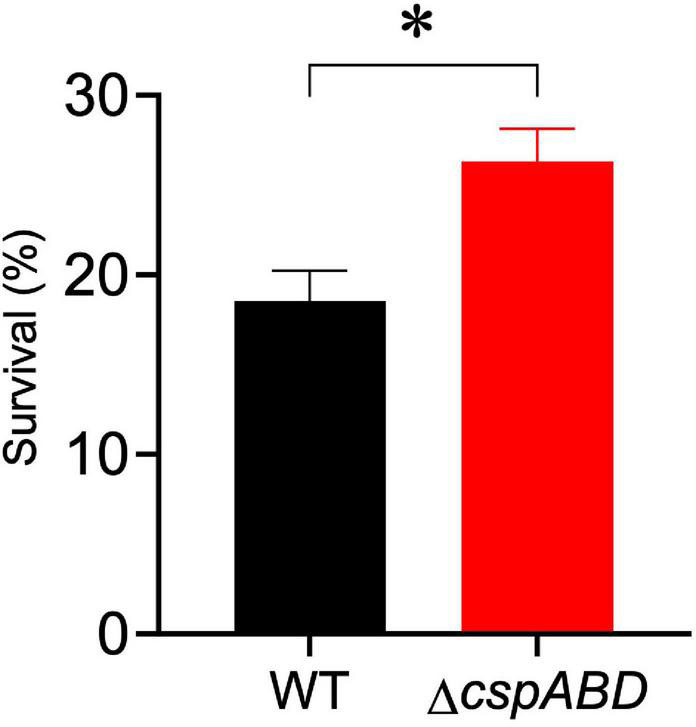
Nisin stress survival comparison between *L. monocytogenes EGDe* wild-type (WT) and Δ*cspABD* strains. Stationary-phase cultures were subjected to 7.5 ppm nisin stress in BHI at 37°C for 60 min. The survival for each strain expressed as a percentage was measured as the number of colony-forming units determined after nisin stress exposure normalized to the number of unstressed cells present at the beginning of stress exposure. Results showing the mean and SEM of six replicates representing three independent biological experiments are presented. ^∗^ *P* < 0.05, significant differences between the WT and Δ*cspABD* strains identified using the *t*-test for comparison of independent samples.

We initially examined the functional relevance of *csp* genes in *L. monocytogenes* nisin tolerance by comparing nisin stress growth phenotypes between the WT strain and a Δ*cspABD* mutant of *L. monocytogenes* EGDe. This showed that the elimination of all three *csp* genes severely compromises growth under nisin stress (Figures 1A,B). The growth parameters total AUC capturing overall growth dynamics, LPD, MGR, and final maximum cell density (MD), determined for *L. monocytogenes* EGDe WT and Δ*cspABD* strains in nisin-supplemented BHI and normalized for growth of each strain in normal BHI, were compared. AUC, MGR, and MD comparisons revealed that the Δ*cspABD* mutation caused 12. 7-, 155-, and 66.6-fold reductions in growth efficiency under nisin stress compared to the WT strain (Figures 1C–F). On the other hand, no significant differences were detected between the two strains considering the LPD periods determined following the inoculation of stationary-phase organisms into nisin-supplemented BHI (Figure 1D). We further examined if the Δ*cspABD* mutations impacted *L. monocytogenes* survival capability under nisin stress, showing that the Δ*cspABD* mutant survived slightly better than the WT strain exposed to nisin stress (Figure 2). Thus, the nisin stress growth efficiency reduction observed in the Δ*cspABD* mutant cannot be attributed to the reduced survival capability or prolonged LPD but rather to the growth capability differences arising between the mutant and WT strains after the lag phase. All in all, these observations thus indicated the functional requirement for at least one of the three *csp* genes for optimal nisin stress tolerance expression in *L. monocytogenes*.

### Variable Phenotypic Contribution of Individual *csp* Genes to *L. monocytogenes* Nisin Stress Tolerance

**FIGURE 3:**
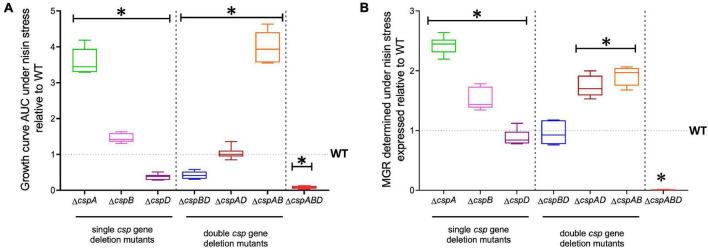
Nisin stress growth efficiency comparison between the wild type (WT) and the single (Δ*cspA*, Δ*cspB*, and Δ*cspD*), double (Δ*cspBD*, Δ*cspAD*, and Δ*cspAB*), and triple (Δ*cspABD*) *csp* deletion mutants of *L. monocytogenes* EGDe. The growth parameters area under the curve **(A)** and maximum growth rate **(B)** under nisin stress normalized to growth in nisin-free BHI were determined for the *csp* mutants and expressed relative to the WT strain levels (WT strain levels equivalent to 1.0 are denoted by a broken line on the graph). The results presented are based on three independent experiments that were performed in duplicates. ^∗^*P* < 0.05, significant differences compared to the WT detected using Tukey’s *post-hoc* test pairwise comparison following one-way ANOVA.

We next sought to distinguish the nisin stress resistance phenotypic contribution roles of the individual *csp* genes. Nisin stress growth phenotypic comparisons based on AUC and MGR in single *csp* gene deletion mutants revealed increased nisin resistance in Δ*cspA* and Δ*cspB* mutants, while the Δ*cspD* mutant had decreased resistance relative to the WT strain (Figure 3). The nisin stress growth phenotype exhibited by the Δ*cspD* mutant was, however, above that of the Δ*cspABD* mutant without any *csp* genes (Figure 3). This observation thus indicates residual nisin stress mitigation from the phenotypic contributions of the intact *csp*A and *cspB* genes that remain within such a single *cspD* gene deletion mutant background. An overall hierarchical trend of Δ*cspA* > Δ*cspB* > Δ*cspD* was thus observed, considering the different nisin stress growth dynamics determined for the three single *csp* gene deletion mutants (Figure 3 and Supplementary Figure 1)—an outcome which indicates redundancy and variable phenotypic contributions between the individual *csp* genes during the survival and growth of *L. monocytogenes* under nisin stress. The *csp* gene phenotypic roles in nisin stress tolerance thus seem epistatic, with nisin resistance phenotype expression seemingly being curbed in the presence of *cspA* and *cspB* functions as their individual deletions increase the expression of nisin phenotypic resistance compared to the WT strain levels (Figure 3).

The nisin stress growth phenotypic consequences observed in single *csp* gene deletion mutants are therefore also mitigated through functional redundancy from the remaining *csp* genes. To assess the phenotypic role of individual *csp* genes without these redundancy influences, nisin stress growth phenotypes were also examined in double *csp* gene deletion mutants expressing single *csp* genes: Δ*cspBD* (expressing *cspA*), Δ*cspAD* (expressing *cspB*), and Δ*cspAB* (expressing *cspD*). The nisin growth phenotypes observed among these single *csp* gene-expressing mutants were, in all cases, superior to a Δ*cspABD* mutant without *csp* genes but varied depending on the remaining *csp* gene (Figure 3). Once again, an overall hierarchical trend of *cspD* (Δ*cspAB*) > *cspB* (Δ*cspAD*) > *cspA* (Δ*cspBD*) was exhibited in nisin stress growth fitness phenotypes based on the observed growth curve AUCs. Notably, the expression of *cspD* alone in Δ*cspAB* increased the nisin stress growth fitness to levels even superior than those of the WT strain (Figure 3 and Supplementary Figure 1)—an observation further supporting the negative regulatory or competitive functional interaction effects associated with CspA and CspB expression that ultimately reduces the expression of nisin stress protection-associated phenotypic responses. The expression of *cspB* alone in the Δ*cspAD* mutant enabled an overall nisin stress growth fitness (AUC) similar to the WT, although it exhibited a faster growth rate under nisin stress than in the WT (Figure 3). On the other hand, *cspA* expression alone (Δ*cspBD*) showed the least contribution to the nisin growth phenotype. Notably, nisin stress growth phenotypic trends exhibited in single *csp* expression backgrounds confirm the hierarchy of *cspD* > *cspB* > *cspA* with respect to their phenotypic contribution to *L. monocytogenes* nisin growth fitness. Moreover, similar phenotypic complementation trends were also observed when the individual *csp* genes were re-introduced into the Δ*cspABD* mutant background through complementation. EGDe_Δ*cspABD*, *cspA* (Δ*cspABD*::pPL2-*cspA*), and *cspB* (and Δ*cspABD*::pPL2-*cspB*)-complemented strains showed a lower nisin growth phenotype restoration, whereas *cspD* (Δ*cspABD*::pPL2-*cspD*) complementation showed the highest level of nisin resistance phenotypic restoration, but the levels achieved in this case were not higher than those of the WT strain (Supplementary Figure 2). Meanwhile, complementing *cspA* in the Δ*cspA* background (Δ*cspA*::pPL2-*cspA*) restored nisin sensitivity to the WT phenotypic level. These observations thus confirmed the involvement of Csps in nisin stress protection responses of *L. monocytogenes*. We also further assessed if *cspD* expressed alone would also be advantageous in the face of dual stress of cold (8°C) and nisin (5 ppm). However, Δ*cspAB*, Δ*cspABD*, and the *cspD*-complemented Δ*cspABD* mutant strain were unable to grow under dual stress, while the WT strain grew, albeit to a significantly reduced extent under such dual stress conditions (Supplementary Figure 3). In addition, the cold growth benefits conferred by *cspA* were diminished in the presence of nisin stress (Supplementary Figure 3). This observation is indicative of the synergistic effects between cold stress and nisin which nullify stress tolerance benefits conferred through *cspD* expression alone. In summary, we established through these analyses the variable phenotypic roles for the three individual *csp* genes toward nisin stress resilience in *L. monocytogenes*. In addition to functional redundancy, the individual *csp* genes also seem to functionally influence the contribution of each other to the expression of nisin stress protection responses in this bacterium.

### Expression Activation of Individual *csp* Genes in Response to Nisin Stress Varies

**FIGURE 4:**
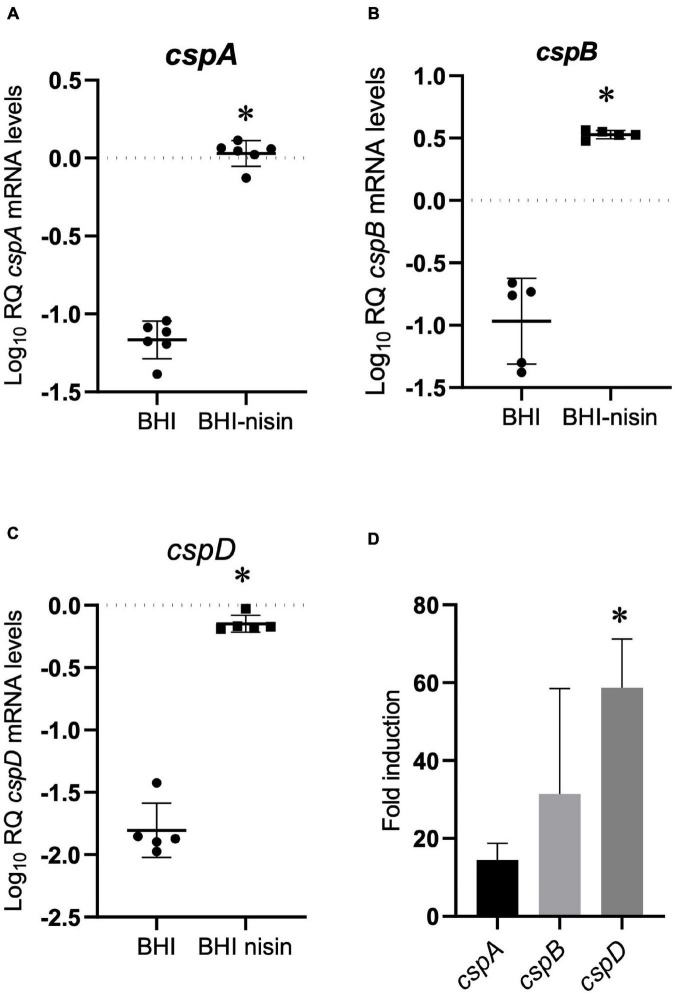
Growth under nisin stress variably induces *csp* mRNA levels. **(A–C)** Relative *csp* mRNA amounts were determined for exponentially growing *L. monocytogenes* EGDe cells cultivated in normal and nisin (5 ppm)-supplemented BHI. The data are presented as scatter plots showing the mean and SD (number of independent biological replicates = 3). 16S rRNA was used for normalization. **(D)** Bar charts showing the fold induction of different *csp* mRNAs relative to the abundance under nisin stress (BHI-nisin) with respect to control levels observed without stress (BHI). ^∗^*P* < 0.05, significant differences in mRNA levels and fold induction identified using the **(A–C)** *t*-test for independent samples and **(D)** Tukey’s *post-hoc* test pairwise comparison following one-way ANOVA.

To examine for a molecular mechanism link between Csps and nisin stress responses, we applied RT-qPCR and assessed the impact of nisin stress exposure on *csp* gene expression. This revealed a variable but significant induction in mRNA abundance for all three *csp* genes when *L. monocytogenes* EGDe cells cultivated under nisin stress in BHI were compared to similarly cultivated control cells without stress (Figures 4A–C). By comparing the nisin stress-associated fold induction trends of *csp* mRNAs, it was shown that the induction magnitude trend reflected nisin stress growth phenotypic relevance observed from the evaluation of single *csp* gene deletion (Δ*cspA* > Δ*cspB* > Δ*cspD*) and single *csp* gene expression (Δ*cspAB* > Δ*cspAD* > Δ*cspBD*) mutants since the nisin-dependent mRNA fold induction levels determined also showed the *cspD* > *cspB* > *cspA* hierarchical trend (Figure 4D).

### Csp Deficiency Increases *L. monocytogenes* Sensitivity to Other Cell Envelope-Targeting Stressors

**FIGURE 5:**
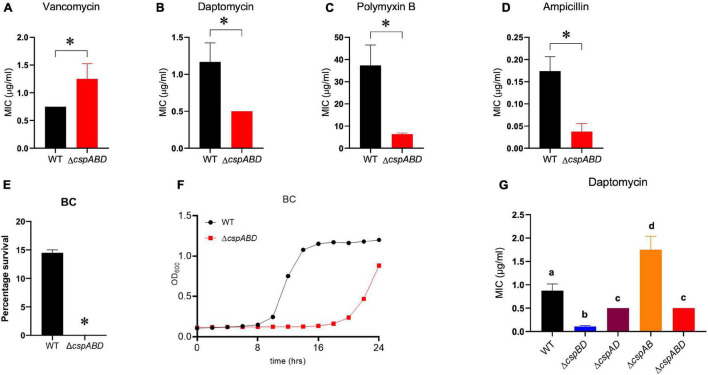
Impact of Csp deficiency on BC and antibiotic susceptibility of *L. monocytogenes*. Comparison of vancomycin, daptomycin, polymyxin B, ampicillin, and BC stress sensitivities between EGDe wild type (WT) and triple (Δ*cspABD*) and double (Δ*cspBD*, Δ*cspAD*, and Δ*cspAB*) *csp* deletion mutants. The minimum inhibitory concentrations (MICs) for vancomycin **(A)**, daptomycin **(B,G)**, polymyxin B **(C)**, and ampicillin **(D)** were determined using the *E*-test. **(E)** WT and Δ*cspABD* strains were exposed BC (10 ppm) at 25°C for 15 min, and survival expressed as a percentage was measured as the number of colony-forming units determined after BC exposure normalized to the number of unstressed cells present at the beginning of stress exposure. **(F)** Growth of WT and Δ*cspABD* strains in BC (1.2 ppm)-supplemented BHI. Results are based on three independent experiments performed in duplicates. ^∗^*P* < 0.05, statistically significant differences between WT and Δ*cspABD* mutant identified using the *t*-test for independent samples. Different letters indicate significant differences between WT and the *csp* mutants daptomycin MICs that were identified using one-way ANOVA and Tukey *post-hoc* test pairwise comparison of all the strains (*p* < 0.05).

The impact of Csp deficiency on *L. monocytogenes* sensitivity to other cell envelope-targeting stressors besides nisin was also examined. Despite displaying a slight increase in resistance to vancomycin, the Δ*cspABD* mutant was more sensitive to cell membrane-targeting cationic peptides daptomycin and polymyxin B (Figures 5A–C). In addition, the Δ*cspABD* mutation also has increased sensitivity to ampicillin, a peptidoglycan synthesis inhibitor targeting penicillin-binding proteins (PBPs) (Figure 5D). The susceptibility of *L. monocytogenes* to the quaternary ammonium compound (QAC) detergent BC was increased without *csp* genes, as the Δ*cspABD* mutant displayed diminished survival and growth efficiency compared to the WT strain under BC stress (Figures 5E,F). Further assessment of daptomycin sensitivity on the other *csp* mutants also revealed that single *csp*-expressing mutants had increased sensitivity than WT, except for Δ*cspAB*-expressing *cspD* that had reduced sensitivity (Figure 5G). An overall hierarchical trend *cspD* (Δ*cspAB*) > *cspB* (Δ*cspAD*) > *cspA* (Δ*cspBD*) in sensitivity to daptomycin was observed similar to the sensitivity trends exhibited under nisin stress. The expression of *cspD* alone (Δ*cspAB*) also increased the daptomycin minimum inhibitory concentration to levels above the WT strain levels. Interestingly, Δ*cspAD*-expressing *cspB* only showed sensitivity comparable to Δ*cspABD*, while the expression of *cspA* alone (Δ*cspBD*) further increased the sensitivity beyond that observed with the *csp* null mutant (Δ*cspABD*)—an observation indicating that CspA might negatively impact Csp-dependent and Csp-independent stress response systems, further compromising the tolerance responses to daptomycin. Overall, these results therefore showed that Csp deficiency generally increases the cell envelope stress susceptibility of *L. monocytogenes* Δ*cspABD* mutant cells, suggesting that Csps contribute toward optimal cell envelope structure constitution and protective barrier functions in this bacterium.

### Impact of Csp Deficiency on the Expression of Nisin Resistance-Associated Genes

**FIGURE 6:**
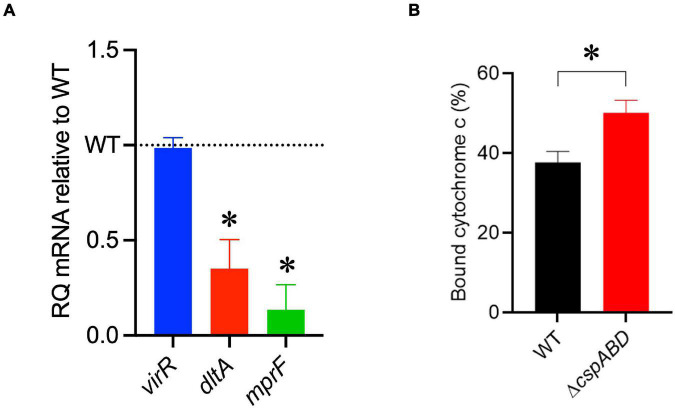
A Δ*cspABD* mutation reduces the mRNA abundance of nisin response genes and cell surface anionic charge. **(A)** *virR*, *dltA*, and *mprF* mRNA levels in *L. monocytogenes* EGDe_Δ*cspABD* are expressed relative to the wild type (WT) strain (WT level is 1.0 and is denoted by a dotted line). RT-qPCR was performed on mRNA isolated from exponentially growing cells cultivated in nisin (1.5 ppm)-supplemented BHI broth. 16S rRNA was used for mRNA normalization. **(B)** Bar graph showing the cytochrome C binding level of exponentially growing EGDe_Δ*cspABD* and WT cells cultivated until OD_600_ 1.0 in BHI at 37°C and 150 rpm. Cytochrome C binding expressed as a percentage was measured as the mean OD_530_ values from replicate samples containing bacteria relative to the mean value of the sample supernatant lacking bacteria. ^∗^*P* < 0.05, statistically significant differences between WT and Δ*cspABD* mutant identified using the *t*-test for independent samples.

**FIGURE 7:**
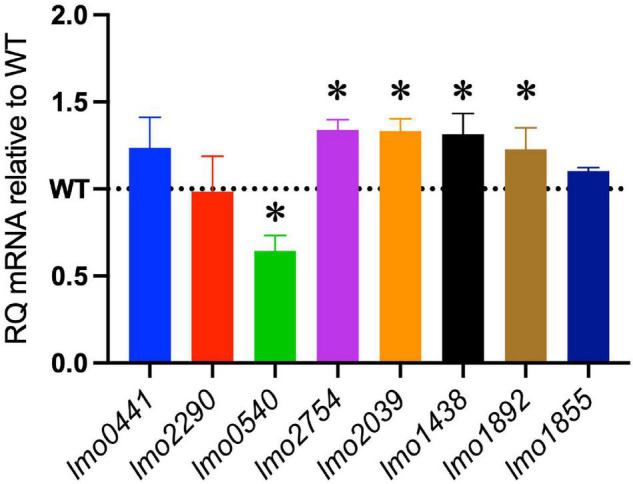
Impact of the Δ*cspABD* mutation on mRNA abundance of selected penicillin-binding protein (PBP) genes. Bar graphs showing the relative quantities of PBP gene mRNAs in EGDe_Δ*cspABD* relative to the WT strain whose level is denoted by a dotted line in stationary-phase cells cultivated in BHI for 16 h at 37°C and 150 rpm. RT-qPCR was performed on mRNA samples isolated from cells cultivated for 16 h at 37°C and 150 rpm in BHI. 16S rRNA was used for mRNA normalization. ^∗^*P* < 0.05, significant differences in mRNA levels between the WT and Δ*cspABD* strains identified using the *t*-test for comparison of independent samples.

The increased sensitivity of the Δ*cspABD* mutant to nisin and other cell envelope stressors pointed toward Csp deficiency-induced changes in stress protective and barrier functions of cell envelope structures. To assess the possible mechanistic links between Csp deficiency and cell envelope structural and functional alteration, the impact of Csp loss on expression regulation of different cell envelope modification-associated genes linked to nisin resistance was examined. RT-qPCR was performed on RNA isolated from exponential-growth-phase cultures cultivated under nisin stress, and the mRNA levels between the WT and Δ*cspABD* strains were compared. Of the two component regulatory systems examined, *liaR* mRNA was upregulated in the Δ*cspABD* mutant, whereas the *virR*, *lisK*, and *cesR* mRNA levels remained similar to those of the WT strain (Supplementary Figure 4). The Δ*cspABD* mutant additionally expressed higher *anrB* (4.46-fold) and similar *telA* mRNA compared to the WT strain (Supplementary Figure 4). On the other hand, despite the unchanged *virR* mRNA expression levels, the Δ*cspABD* mutant contained lower amounts of *dltA* (2.84-fold) and *mprF* (7.4-fold) mRNAs, both of which are positively regulated through VirR, than the WT strain (Figure 6A). The *dltABCD* operon and *mprF* gene products contribute toward the net cell envelope positive charge through cell wall (WTA D-alanylation) and cell membrane (lysinylation) modification, respectively. Thus, a possible consequence of low *dltA* and *mprF* expression would be a more electronegatively charged cell envelope. Cytochrome c binding comparison was used as a measure of cell surface net charge, showing that the Δ*cspABD* mutant indeed has a more electronegative cell surface as its cells bound more cytochrome c than the WT strain (Figure 6B). Cell WTA L-rhamnosylation, which is mediated by the rhamnosyltransferase RmlT, is another stress-protective cell envelope modification strategy that reduces the access of antimicrobial peptides to the cell membrane. We assessed if this modification might be altered without Csps by comparing the *rmlT* mRNA expression between Δ*cspABD* and WT strains cultivated in minimal media with L-rhamnose as the sole carbon source. This showed that the Δ*cspABD* expressed lower *rmlT* mRNA levels than the WT strain during growth on L-rhamnose—an observation that suggests that WTA rhamnosylation might be reduced without Csps contributing to increased cell membrane vulnerability to antimicrobial peptides, including nisin (Supplementary Figure 5). Finally, the increased sensitivity of the Δ*cspABD* mutant to ampicillin also points toward Csp deficiency-altering PBP expression and activity. The altered expression balance and activity of PBPs can induce peptidoglycan synthesis, composition, and structural modification changes. Upon comparing transcripts of selected PBP genes in stationary-phase-growth-stage *L. monocytogenes* cells cultivated in BHI, it was shown that there was reduced abundance in mRNA associated with PBP Lmo0540, whereas those for PBPs Lmo2754, Lmo2309, Lmo1438, and Lmo1892 were increased in the Δ*cspABD* mutant compared to the WT strain (Figure 7). Overall, our analysis of gene expression suggests that Csp deficiency might increase nisin sensitivity through altered cell membrane and cell wall synthesis and modification processes, thus causing a more electronegative cell surface and possibly an altered peptidoglycan composition, structure, and function.

## Discussion

Nisin is a widely used bacteriocin for mitigation against food-associated pathogenic and spoilage bacteria (Bali et al., 2016; Gharsallaoui et al., 2016; Santos et al., 2018; Ibarra-Sánchez et al., 2020; Martinez-Rios et al., 2021). The efficacy of nisin against *L. monocytogenes* is, however, currently reduced through various intrinsic nisin resistance molecular response systems, most of which we do not yet fully understand (Gravesen et al., 2002; Bergholz et al., 2013; Zhou et al., 2014; Draper et al., 2015; Bucur et al., 2018; Wambui et al., 2020; Pinilla et al., 2021). An improved understanding of these mechanisms will thus enhance our capability to design better strategies for nisin deployment against this pathogen. The function of Csps is crucial in global gene expression regulatory events underlying the normal growth physiological responses as well as virulence and stress protection phenotypes in bacteria (Keto-Timonen et al., 2016; Muchaamba et al., 2021). Studies by others have previously shown that, when exposed to nisin stress, *L. monocytogenes* also responds through activation of *csp* gene expression (Liu et al., 2013; Wu et al., 2018). Such observations suggest that global regulatory functions through Csps might contribute to stress protection responses required for the survival and growth of this bacterium in the presence of nisin. In the current study, the functional requirements for Csps in *L. monocytogenes* nisin stress tolerance were examined, which revealed that Csp regulatory inputs were indeed essential for the full expression of intrinsic nisin stress protection responses in *L. monocytogenes*. This seems to be achieved through mechanisms that at least, in part, involve Csp-dependent regulatory input that promotes the optimal expression of cell envelope-associated modification and stress protection functions.

We initially showed that the removal of all the three *csp* genes severely compromises nisin stress resilience, strongly attenuating growth in a *L. monocytogenes* EGDe Δ*cspABD* mutant cultivated under nisin stress conditions. The functional contributions of individual *csp* genes to nisin stress tolerance in this bacterium were distinguished through the phenotypic analysis of single *csp* gene-deleted mutants (Δ*cspA*, Δ*cspB*, and Δ*cspD*) as well as double *csp* gene-deleted mutants (Δ*cspBD*, Δ*cspAD*, and Δ*cspAB*) expressing single *csp* genes. This confirmed that functional inputs from all the three *csp* genes are necessary for the optimal expression of the nisin stress tolerance phenotype in this bacterium. Nonetheless, despite sharing some functionally redundant roles, the contributions of the three *csp* genes toward the nisin stress tolerance phenotype were not equal but rather displayed a clear hierarchical trend—*cspD* > *cspB* > *cspA*—with respect to their ability to promote the resistance and growth of *L. monocytogenes* under nisin stress.

We found that, in addition to increasing nisin stress vulnerability, Csp function deficiency also increases *L. monocytogenes* susceptibility to other cell envelope-targeting stressors. Csp loss increased the sensitivity to other membrane-active cationic antimicrobial peptides such as daptomycin and polymyxin B as well as BC, a membrane-active cationic detergent widely used for disinfection in hospital settings and food production environments. The lack of Csps additionally increased the susceptibility of *L. monocytogenes* to peptidoglycan synthesis inhibition through the β-lactam antibiotic ampicillin. Overall, these phenotypic defects thus indicate that consequences of Csp function loss include cell envelope structural and functional changes that increase the sensitivity to both cell wall- and cell membrane-targeting stress. Bacteria cell envelopes, among other functions, are also important in sensing and adaptation against cold and salt stress conditions (Jordan et al., 2008; Silhavy et al., 2010; Bergholz et al., 2012; Paul, 2013; Asmar et al., 2017; Bucur et al., 2018). Thus, a defective cell envelope structure and function in a Δ*cspABD* mutant would be consistent with previous observations that such a mutation increases *L. monocytogenes* sensitivity to these food-relevant stress conditions (Schmid et al., 2009).

Our observations on the ability of individual *csp* genes to restore nisin tolerance phenotype when expressed alone revealed variable functional roles as well as suggested complex functional and regulatory network interactions between the individual *csp* genes in view of their phenotypic contribution to *L. monocytogenes* nisin stress tolerance. Notably, the individual deletion of *cspA* as well as *cspB* to a lesser extent induced increased resistance and growth of *L. monocytogenes* under nisin stress. One possible explanation for this observation is that the expression and activities of *cspA* and *cspB* under the applied experimental conditions might be energetically costly, thus their inactivation avails more cellular resources for stress protection responses, allowing more efficient growth under nisin stress. Alternatively, these *csp* genes might also have negative regulatory effects that keep the activity of other Csps, such as CspD activity, in check. Consequently, their removal enhances the expression of CspD activities, including nisin stress protective functions. The latter hypothesis is supported by the fact that we found that expressing *cspD* alone conferred the highest levels of nisin stress tolerance, enabling even more efficient adaptation and growth under nisin stress to levels that even surpassed the WT strain. Along these lines, the upregulation of *cspD* and *cspB* mRNA levels were previously detected in *cspA* deletion mutants compared to their WT strains in response to desiccation stress (Kragh et al., 2020). Furthermore, studies in bacteria such as *E. coli* and *S. aureus* have also shown that Csps can have positive and negative regulatory effects on their own expression and that of other Csps (Bae et al., 1999; Caballero et al., 2018). The loss of *cspA* and *cspB* might inadvertently induce general stress resistance in mutated organisms due to stressful cellular conditions arising from the loss of CspA and CspB functions, which might lead to a general increase in nisin stress tolerance compared to the WT strain.

Nisin targets *L. monocytogenes* through a mechanism that disrupts peptidoglycan and cell membrane synthesis and homeostasis (Bruno et al., 1992; Abee et al., 1994; Wiedemann et al., 2001). Previous studies have shown that cell wall and cell membrane changes are associated with altered nisin sensitivity in *L. monocytogenes* (Gravesen et al., 2004; Zhou et al., 2014; Bucur et al., 2018). Our observations here thus indicated that the Csp function deficiency might induce cell envelope changes that increase the vulnerability to cell wall- and cell membrane-targeting stressors such as nisin. D-Alanylation of cell wall teichoic acids and membrane phospholipid lysinylation are well-known cell envelope modifications that protect against nisin stress in *L. monocytogenes*. These processes involve the function of *dltABCD* operon and *mprF* gene products, respectively (Bucur et al., 2018). The expression of these protein systems is, in part, regulated through the VirRS two-component regulatory system (Kang et al., 2015; Grubaugh et al., 2018). Monitoring the levels of mRNA transcripts derived from these two loci showed that Csp deficiency reduced expression from the *dltABCD* operon (*dltA*) and *mprF* genes but had no impact on *virR* expression in *L. monocytogenes* cells cultivated under nisin stress. One possible explanation is that Csps influenced the *dltA* and *mprF* mRNA levels downstream of VirR regulation. This might be due to loss of Csp-associated transcription activation, antitermination, and mRNA stability functions (Bae et al., 2000; Phadtare et al., 2002; Phadtare and Severinov, 2010, 2016; Holmqvist and Vogel, 2018). We have previously demonstrated that this Δ*cspABD* mutation results in reduced listeriolysin O protein production, in part due to the reduced transcripts and the low stability of *hly* mRNA encoding for this protein (Schärer et al., 2013). Alternatively, as seen with CspR in *Enterococcus faecalis*, the effects of Csp absence on *virR* might be observable at the posttranscriptional level, having similar or more mRNA transcripts but less protein; however, such a possibility remains to be experimentally confirmed (Michaux et al., 2012). Eshwar et al. (2017) showed that *csp* mutants contained increased *actA* and *flaA* mRNA transcripts compared to the WT strain but had reduced or completely lacked ActA proteins and flagella, suggesting Csp contribution in post-transcriptional regulation of these genes. *L. monocytogenes* Δ*cspABD* mutant cells containing lower *dltA* and *mprF* mRNA levels are therefore expected to possess a more electronegative cell surface with an increased capacity to bind positively charged cytochrome c molecules. The increased cell envelope stress sensitivity to cationic antimicrobial peptides, such as nisin and the positive QAC detergent BC, observed upon the loss of Csps can thus, in part, be explained by the more anionic cell envelope induced through the loss of Csp-dependent regulation on Dlt and MprF protein-associated cell wall and cell membrane modifications. This disruption of native DltA and MprF protein regulation due to Csp loss thus hinders the ability of *L. monocytogenes* to respond to different stressors through cell envelope modification processes involving peptidoglycan D-alanylation and membrane phospholipid lysinylation.

Besides the altered peptidoglycan modification, the loss of Csps could also contribute to an altered peptidoglycan constitution through the loss of their regulatory inputs on PBP expression. The increased expression of PBPs, such as PBP2229 (Lmo2229), has previously been shown to confer or alter nisin resistance in *L. monocytogenes* (Gravesen et al., 2001, 2004). In this study, we observed a downregulation of PBP gene *lmo0540* in the WT strain, while PBPs encoding genes *lmo2754*, *lmo2309*, *lmo1438*, and *lmo1892* were upregulated in the Δ*cspABD* mutant compared to the WT strain. Such differential gene expression between the WT and mutant strains could have contributed to the observed phenotypic differences under nisin stress. The peptidoglycan structure and content of Δ*cspABD* might have favored survival while impairing growth as observed with its slightly better survival compared to the WT but overall compromised growth under nisin stress.

Regulatory systems, such as CesR, LisRK, LiaRS, VirRS, and sigma factors, are critical for fine-tuning the responses against stressors, such as nisin and cell envelop-targeting antibiotics (Cotter et al., 2002; Mascher et al., 2004; Mandin et al., 2005; Bucur et al., 2018), while ABC transporters also play important roles by removing these stressors from the cell envelope (Lubelski et al., 2006; Velamakanni et al., 2008; Collins et al., 2010a). Among the analyzed two-component system genes, only *liaR* was significantly upregulated in Δ*cspABD*. This upregulation of the LiaRS two component system, coupled with the increased expression of *anrB*, might have contributed to the reduced sensitivity of the mutant toward vancomycin compared to the WT strain. The increased sensitivity of Δ*cspABD* to ampicillin, yet being less sensitive to vancomycin, might be related to differences in the mechanism of action of these two antibiotics. Ampicillin mainly interferes with peptidoglycan synthesis by binding to PBPs inhibiting transpeptidation, while vancomycin prevents cell wall cross-linking by binding to the acyl-D-Alanine-D-Alanine portion of the growing cell wall (Watanakunakorn, 1984; Kaushik et al., 2014). Resistance to vancomycin in other bacteria, such as enterococci, has been linked to the alteration of the peptidoglycan synthesis pathway, involving the substitution of D-alanine-D-alanine to either D-alanine-D-lactate or D-alanine-D-serine (Ahmed and Baptiste, 2018). These changes significantly reduce vancomycin binding. Similar changes to peptidoglycan synthesis pathways might have occurred in the mutant, or they might have been structural changes brought about by the differential expression of PBPs, making the site of action of vancomycin less accessible and hence the observed increased tolerance. Similarly, the stationary-phase cells of Δ*cspABD* mutant had greater survival under nisin (7.5 ppm) stress compared to the WT strain. The reason for this increased survival is not yet clear. We can only speculate that the cell envelope and the general cell physiological state of the mutant at this growth stage improve its survival responses against nisin. Upregulation of genes, such as *liaR* and *anrB*, in the Δ*cspABD* mutant might be contributing to this phenotype. However, this needs to be experimentally validated.

Previous work by others demonstrated that decoration of WTA with L-rhamnose increases bacterial resistance to AMPs by delaying their interaction and disruption of the plasma membrane, thereby promoting *L. monocytogenes in vivo* survival and pathogenicity (Carvalho et al., 2015). Our observation that Csps absence results in lower *rmlT* transcripts, which encodes a crucial effector for WTA L-rhamnose glycosylation, provides another pathway by which Csps might further influence nisin stress sensitivity. Decreased WTA rhamnosylation would increase the accessibility of the cell membrane, rendering it more susceptible to nisin stress. In the context of host pathogenicity, these findings are suggestive that nisin-stressed WT *L. monocytogenes* cells responding to this stress through increased L-rhamnosylation can inadvertently be primed for evasion and tolerance of host AMP-mediated defenses.

Cross-protection for many stress conditions and hurdle procedures, including nisin, has been reported in *L. monocytogenes* and other foodborne pathogens (Bergholz et al., 2012, 2013; Kaur et al., 2013; Begley and Hill, 2015; Malekmohammadi et al., 2017; Abeysundara et al., 2019; Chen et al., 2020; Henderson et al., 2020; Wu et al., 2021). Since *csp* genes are induced across most of these hurdle procedures, they might act, in part, as mediators of this cross-protection—for instance, CspD, which is critical for growth under osmotic stress, is also critical for nisin stress tolerance, and thus its induction by one of these stresses might inadvertently induce cross-protection to the other (Schmid et al., 2009). The phenomenon that Csp functions can be inhibitory to each other in some situations is encouraging; conditions that induce the inhibitory Csp might increase *L. monocytogenes* sensitivity to a stressor that requires the functions of the inhibited Csp. We show here that, under dual cold and nisin stress conditions, the Δ*cspAB* mutant expressing *cspD* alone and showing superior growth, compared to the WT strain, under nisin stress alone completely loses its growth ability. On the other hand, the WT is still able to grow under these dual stress conditions, albeit at a slower rate than that observed under cold stress alone. Moreover, both Δ*cspBD* and Δ*cspABD*::pPL2-*cspA* expressing *cspA* alone that grow under cold stress alone also lost cold growth ability when grown under dual cold and nisin stress. These observations, especially with the WT, are suggestive that the combination of hurdle techniques with opposing Csp requirements might potentiate inhibition efficacy, thereby increasing food safety. Thus, such effects must be considered when combining hurdle techniques or using them in series.

In the present study, the effects of Csp absence on pathways known to be involved in nisin stress responses and against other cell envelope-targeting stressors were investigated (Liu et al., 2013; Bucur et al., 2018; Wu et al., 2018; Pinilla et al., 2021). However, such an approach might miss other novel pathways contributing to the differences between the *csp* deletion mutants and the WT strains under nisin stress. A global approach which includes full transcriptome and proteome analysis must therefore be employed in future studies.

In conclusion, we have shown that Csps are crucial for stress tolerance against the food preservative nisin. Their absence increases sensitivity to cell wall- and cell membrane-targeting antimicrobials and chemicals. At the mechanistic level, we showed that Csp deficiency reduces the expression of genes encoding cell envelope-modifying proteins such as *dltA* and *mprF*. The consequences of such changes included increased electronegativity of the cell envelop in *csp* deletion mutants, thus reducing the electrostatic repulsion of cationic antimicrobials. We thus presume that the increased cell envelope stress sensitivity observed upon the loss of Csps is, in part, due to the loss of their regulatory effect on the expression of important cell wall and cell membrane modification proteins. By disrupting the native regulation of the DltA and MprF proteins, Csp loss hinders the ability of *L. monocytogenes* to respond to different cell wall- and cell membrane-targeting stressors requiring stress responses mediated through D-alanylation and lysinylation of cell WTA and membrane phospholipids, respectively. Overall, our study shows that Csps play important roles in *L. monocytogenes* survival and transmission in food and different processing environments. Such knowledge can be applied to improve food safety, ensuring hurdle techniques to avoid unintended cross-protection induction which might nullify otherwise effective interventions.

## Data Availability Statement

The original contributions presented in the study are included in the article/Supplementary Material, further inquiries can be directed to the corresponding author/s.

## Author Contributions

TT contributed to the conceptualization and supervision. JW and FM contributed to the investigation and the writing—original draft preparation. RS, FM, JW, and TT contributed to the writing—review and editing. RS contributed to the funding acquisition. All authors read and approved the final manuscript.

## Conflict of Interest

The authors declare that the research was conducted in the absence of any commercial or financial relationships that could be construed as a potential conflict of interest.

## Publisher’s Note

All claims expressed in this article are solely those of the authors and do not necessarily represent those of their affiliated organizations, or those of the publisher, the editors and the reviewers. Any product that may be evaluated in this article, or claim that may be made by its manufacturer, is not guaranteed or endorsed by the publisher.

## References

[B1] Abachin E. Poyart C. Pellegrini E. Milohanic E. Fiedler F. Berche P. (2002). Formation of D-alanyl-lipoteichoic acid is required for adhesion and virulence of *Listeria monocytogenes*. *Mol. Microbiol.* 43 1–14. 10.1046/j.1365-2958.2002.02723.x 11849532

[B2] Abee T. Rombouts F. M. Hugenholtz J. Guihard G. Letellier L. (1994). Mode of action of nisin Z against *Listeria monocytogenes* ScottA grown at high and low temperatures. *Appl. Environ. Microbiol.* 60 1962–1968. 10.1128/aem.60.6.1962-1968.1994 16349286 PMC201587

[B3] Abeysundara P. D. A. Dhowlaghar N. Nannapaneni R. (2019). Influence of cold stress on the survival of *Listeria monocytogenes* Bug600 and ScottA in lethal alkali, acid and oxidative stress. *Lebensm. Wiss. Technol.* 100 40–47.

[B4] Ahmed M. O. Baptiste K. E. (2018). Vancomycin-Resistant enterococci: a review of antimicrobial resistance mechanisms and perspectives of human and animal health. *Microb. Drug Resist.* 24 590–606. 10.1089/mdr.2017.0147 29058560

[B5] Alvarez-Sieiro P. Montalbán-López M. Mu D. Kuipers O. P. (2016). Bacteriocins of lactic acid bacteria: extending the family. *Appl. Microbiol. Biotechnol.* 100 2939–2951. 10.1007/s00253-016-7343-9 26860942 PMC4786598

[B6] Asmar A. T. Ferreira J. L. Cohen E. J. Cho S.-H. Beeby M. Hughes K. T. (2017). Communication across the bacterial cell envelope depends on the size of the periplasm. *PLoS Biol.* 15:e2004303. 10.1371/journal.pbio.2004303 29257832 PMC5736177

[B7] Bae W. Phadtare S. Severinov K. Inouye M. (1999). Characterization of *Escherichia coli* cspE, whose product negatively regulates transcription of cspA, the gene for the major cold shock protein. *Mol. Microbiol.* 31 1429–1441. 10.1046/j.1365-2958.1999.01284.x 10200963

[B8] Bae W. Xia B. Inouye M. Severinov K. (2000). *Escherichia coli* CspA-family RNA chaperones are transcription antiterminators. *Proc. Natl. Acad. Sci. U.S.A.* 97 7784–7789. 10.1073/pnas.97.14.7784 10884409 PMC16622

[B9] Bali V. Panesar P. S. Bera M. B. Kennedy J. F. (2016). Bacteriocins: recent trends and potential applications. *Crit. Rev. Food Sci. Nutr.* 56 817–834. 10.1080/10408398.2012.729231 25117970

[B10] Begley M. Hill C. (2015). Stress adaptation in foodborne pathogens. *Annu. Rev. Food Sci. Technol.* 6 191–210. 10.1146/annurev-food-030713-092350 25665171

[B11] Begley M. Cotter P. D. Hill C. Ross R. P. (2010). Glutamate decarboxylase-mediated nisin resistance in *Listeria monocytogenes*. *Appl. Environ. Microbiol.* 76 6541–6546. 10.1128/AEM.00203-10 20693450 PMC2950483

[B12] Begley M. Hill C. Ross R. P. (2006). Tolerance of *Listeria monocytogenes* to cell envelope-acting antimicrobial agents is dependent on SigB. *Appl. Environ. Microbiol.* 72 2231–2234. 10.1128/AEM.72.3.2231-2234.2006 16517678 PMC1393204

[B13] Bergholz T. M. Bowen B. Wiedmann M. Boor K. J. (2012). *Listeria monocytogenes* shows temperature-dependent and -independent responses to salt stress, including responses that induce cross-protection against other stresses. *Appl. Environ. Microbiol.* 78 2602–2612. 10.1128/AEM.07658-11 22307309 PMC3318819

[B14] Bergholz T. M. Tang S. Wiedmann M. Boor K. J. (2013). Nisin resistance of *Listeria monocytogenes* is increased by exposure to salt stress and is mediated via LiaR. *Appl. Environ. Microbiol.* 79 5682–5688. 10.1128/AEM.01797-13 23851083 PMC3754191

[B15] Bruno M. E. C. Kaiser A. Montville T. J. (1992). Depletion of proton motive force by nisin in *Listeria monocytogenes* cells. *Appl. Environ. Microbiol.* 58 2255–2259. 10.1128/aem.58.7.2255-2259.1992 1637163 PMC195764

[B16] Bucur F. I. Grigore-Gurgu L. Crauwels P. Riedel C. U. Nicolau A. I. (2018). Resistance of *Listeria monocytogenes* to stress conditions encountered in food and food processing environments. *Front. Microbiol.* 9:2700. 10.3389/fmicb.2018.02700 30555426 PMC6282059

[B17] Burgess C. M. Gianotti A. Gruzdev N. Holah J. Knøchel S. Lehner A. (2016). The response of foodborne pathogens to osmotic and desiccation stresses in the food chain. *Int. J. Food Microbiol.* 221 37–53. 10.1016/j.ijfoodmicro.2015.12.014 26803272

[B18] Caballero C. J. Menendez-Gil P. Catalan-Moreno A. Vergara-Irigaray M. García B. Segura V. (2018). The regulon of the RNA chaperone CspA and its auto-regulation in *Staphylococcus aureus*. *Nucleic Acids Res.* 46 1345–1361. 10.1093/nar/gkx1284 29309682 PMC5815144

[B19] Carvalho F. Atilano M. L. Pombinho R. Covas G. Gallo R. L. Filipe S. R. (2015). L- Rhamnosylation of *Listeria monocytogenes* wall Teichoic acids promotes resistance to antimicrobial peptides by delaying interaction with the membrane. *PLoS Pathog.* 11:e1004919. 10.1371/journal.ppat.1004919 26001194 PMC4441387

[B20] Centers for Disease Control [CDC] (2018). Centre for disease control and prevention. Preliminary incidence and trends of infections with pathogens transmitted commonly through food. Foodborne Diseases Active Surveillance Network, 10 U.S. Sites, 2006-2017. *MMWR Morb. Mortal. Wkly. Rep.* 67 324–328. 29565841 10.15585/mmwr.mm6711a3 PMC5868202

[B21] Chen R. Skeens J. Orsi R. H. Wiedmann M. Guariglia-Oropeza V. (2020). Pre-growth conditions and strain diversity affect nisin treatment efficacy against *Listeria monocytogenes* on cold-smoked salmon. *Int. J. Food Microbiol.* 333:108793. 10.1016/j.ijfoodmicro.2020.108793 32763758

[B22] Collins B. Curtis N. Cotter P. D. Hill C. Ross R. P. (2010a). The ABC transporter AnrAB contributes to the innate resistance of *Listeria monocytogenes* to nisin, bacitracin, and various beta-lactam antibiotics. *Antimicrob. Agents Chemother.* 54 4416–4423. 10.1128/AAC.00503-10 20643901 PMC2944581

[B23] Collins B. Joyce S. Hill C. Cotter P. D. Ross R. P. (2010b). TelA contributes to the innate resistance of *Listeria monocytogenes* to nisin and other cell wall- acting antibiotics. *Antimicrob. Agents Chemother.* 54 4658–4663. 20713661 10.1128/AAC.00290-10 PMC2976142

[B24] Cotter P. D. Guinane C. M. Hill C. (2002). The LisRK signal transduction system determines the sensitivity of *Listeria monocytogenes* to nisin and cephalosporins. *Antimicrob. Agents Chemother.* 46 2784–2790. 10.1128/AAC.46.9.2784-2790.2002 12183229 PMC127401

[B25] Cotter P. D. Ross R. P. Hill C. (2013). Bacteriocins – a viable alternative to antibiotics? *Nat. Rev. Microbiol.* 11 95–105. 10.1038/nrmicro2937 23268227

[B26] Cruz-Loya M. Kang T. M. Lozano N. A. Watanabe R. Tekin E. Damoiseaux R. (2019). Stressor interaction networks suggest antibiotic resistance co-opted from stress responses to temperature. *ISME J.* 13 12–23. 10.1038/s41396-018-0241-7 30171253 PMC6298959

[B27] Draper L. A. Cotter P. D. Hill C. Ross R. P. (2015). Lantibiotic resistance. *Microbiol. Mol. Biol. Rev.* 79 171–191. 10.1128/MMBR.00051-14 25787977 PMC4394878

[B28] Eshwar A. K. Guldimann C. Oevermann A. Tasara T. (2017). Cold-Shock Domain Family Proteins (Csps) are involved in regulation of virulence, cellular aggregation, and flagella-based motility in *Listeria monocytogenes*. *Front. Cell. Infect. Microbiol.* 7:453. 10.3389/fcimb.2017.00453 29124040 PMC5662587

[B29] European Food Safety Authority [EFSA] (2017). The European Union summary report on trends and sources of zoonoses, zoonotic agents and food-borne outbreaks in 2016. *EFSA J.* 15:5077. 10.2903/j.efsa.2017.5077 32625371 PMC7009962

[B30] Feng Y. Huang H. Liao J. Cohen S. N. (2001). *Escherichia coli* poly(A)- binding proteins that interact with components of degradosomes or impede RNA decay mediated by polynucleotide phosphorylase and RNase E. *J. Biol. Chem.* 276 31651–31656. 10.1074/jbc.M102855200 11390393

[B31] Fritsch F. Mauder N. Williams T. Weiser J. Oberle M. Beier D. (2011). The cell envelope stress response mediated by the LiaFSRLm three-component system of *Listeria monocytogenes* is controlled via the phosphatase activity of the bifunctional histidine kinase LiaSLm. *Microbiology* 157 373–386. 10.1099/mic.0.044776-0 21030435

[B32] Gandhi M. Chikindas M. L. (2007). *Listeria*: a foodborne pathogen that knows how to survive. *Int. J. Food Microbiol.* 113 1–15. 10.1016/j.ijfoodmicro.2006.07.008 17010463

[B33] Gharsallaoui A. Oulahal N. Joly C. Degraeve P. (2016). Nisin as a food preservative: part 1: physicochemical properties, antimicrobial activity, and main uses. *Crit. Rev. Food Sci. Nutr.* 56 1262–1274. 10.1080/10408398.2013.763765 25675115

[B34] Glaser P. Frangeul L. Buchrieser C. Rusniok C. Amend A. Baquero F. (2001). Comparative genomics of Listeria species. *Science* 294, 849–852. 10.1126/science.1063447 11679669

[B35] Göker M. (2016). *Analysing Growth Curves and Other User-Defined Data in Opm. 1–18.* Available online at: http://www.goeker.org/opm/opm_doc/doc/opm-growth-curves.pdf (accessed August 12, 2021).

[B36] Göker M. Hofner B. Montero Calasanz M.d.C. Sikorski J. Vaas L. A. I (2016). *opm: An R Package for Analysing Phenotype Microarray and Growth Curve Data. Phenotype Microarray Data: 1-68.* Available online at: http://www.goeker.org/opm/opm_doc/doc/opm-tutorial.pdf (accessed December 26, 2020).

[B37] Graumann P. Wendrich T. M. Weber M. H. W. Schröder K. Marahiel M. A. (1997). A family of cold shock proteins in *Bacillus subtilis* is essential for cellular growth and for efficient protein synthesis at optimal and low temperatures. *Mol. Microbiol.* 25 741–756. 9379903 10.1046/j.1365-2958.1997.5121878.x

[B38] Gravesen A. Jydegaard Axelsen A. M. Mendes Da Silva J. Hansen T. B. Knochel S. (2002). Frequency of bacteriocin resistance development and associated fitness costs in *Listeria monocytogenes*. *Appl. Environ. Microbiol.* 68 756–764. 10.1128/AEM.68.2.756-764.2002 11823216 PMC126701

[B39] Gravesen A. Kallipolitis B. Holmstrøm K. Høiby P. E. Ramnath M. Knøchel S. (2004). pbp2229-mediated nisin resistance mechanism in *Listeria monocytogenes* confers cross-protection to class IIa bacteriocins and affects virulence gene expression. *Appl. Environ. Microbiol.* 70 1669–1679. 10.1128/AEM.70.3.1669-1679.2004 15006792 PMC368357

[B40] Gravesen A. Sørensen K. Aarestrup F. M. Knøchel S. (2001). Spontaneous nisin-resistant *Listeria monocytogenes* mutants with increased expression of a putative penicillin-binding protein and their sensitivity to various antibiotics. *Microb. Drug Resist.* 7 127–135. 10.1089/10766290152045002 11442339

[B41] Grubaugh D. Regeimbal J. M. Ghosh P. Zhou Y. Lauer P. Dubensky T. W. (2018). The VirAB ABC transporter is required for VirR regulation of *Listeria monocytogenes* virulence and resistance to nisin. *Infect. Immun.* 86:e00901–17. 10.1128/IAI.00901-17 29263107 PMC5820956

[B42] Henderson L. O. Erazo Flores B. J. Skeens J. Kent D. Murphy S. I. Wiedmann M. (2020). Nevertheless, she resisted – role of the environment on *Listeria monocytogenes* sensitivity to nisin treatment in a laboratory cheese model. *Front. Microbiol.* 11:635. 10.3389/fmicb.2020.00635 32328054 PMC7160321

[B43] Holmqvist E. Vogel J. (2018). RNA-binding proteins in bacteria. *Nat. Rev. Microbiol.* 16 601–615. 10.1038/s41579-018-0049-5 29995832

[B44] Hudson W. H. Ortlund E. A. (2014). The structure, function and evolution of proteins that bind DNA and RNA. *Nat. Rev. Mol. Cell Biol.* 15 749–760. 10.1038/nrm3884 25269475 PMC4280011

[B45] Ibarra-Sánchez L. A. El-Haddad N. Mahmoud D. Miller M. J. Karam L. (2020). Invited review: advances in nisin use for preservation of dairy products. *J. Dairy Sci.* 103 2041–2052. 10.3168/jds.2019-17498 31928749

[B46] Jiang X. Geng Y. Ren S. Yu T. Li Y. Liu G. (2019). The VirAB-VirSR-AnrAB multicomponent system is involved in resistance of *Listeria monocytogenes* EGD-e to cephalosporins, bacitracin, nisin, benzalkonium chloride, and ethidium bromide. *Appl. Environ. Microbiol.* 85:e01470–19. 10.1128/AEM.01470-19 31399408 PMC6805085

[B47] Jordan S. Hutchings M. I. Mascher T. (2008). Cell envelope stress response in Gram-positive bacteria. *FEMS Microbiol. Rev.* 32 107–146. 10.1111/j.1574-6976.2007.00091.x 18173394

[B48] Kallipolitis B. H. Ingmer H. Gahan C. G. Hill C. Søgaard-Andersen L. (2003). CesRK, a two-component signal transduction system in *Listeria monocytogenes*, responds to the presence of cell wall-acting antibiotics and affects β-lactam resistance. *Antimicrob. Agents Chemother.* 47 3421–3429. 10.1128/AAC.47.11.3421-3429.2003 14576097 PMC253798

[B49] Kang J. Wiedmann M. Boor K. J. Bergholz T. M. (2015). VirR-mediated resistance of *Listeria monocytogenes* against food antimicrobials and cross- protection induced by exposure to organic acid salts. *Appl. Environ. Microbiol.* 81 4553–4562. 10.1128/AEM.00648-15 25911485 PMC4475887

[B50] Kaur G. Singh T. P. Malik R. K. (2013). Antibacterial efficacy of nisin, pediocin 34 and enterocin FH99 against *Listeria monocytogenes* and cross resistance of its bacteriocin resistant variants to common food preservatives. *Braz. J. Microbiol.* 44 63–71. 10.1590/S1517-83822013005000025 24159285 PMC3804179

[B51] Kaur G. Singh T. P. Malik R. K. Bhardwaj A. (2012). Mechanism of nisin, pediocin 34, and enterocin FH99 resistance in *Listeria monocytogenes*. *Probiotics Antimicrob. Proteins* 4 11–20. 10.1007/s12602-011-9085-4 26781732

[B52] Kaushik D. Mohan M. Borade D. M. Swami O. C. (2014). Ampicillin: rise fall and resurgence. *J. Clin. Diagn. Res.* 8 ME01–ME3. 10.7860/JCDR/2014/8777.4356 24995206 PMC4080027

[B53] Keto-Timonen R. Hietala N. Palonen E. Hakakorpi A. Lindström M. Korkeala H. (2016). Cold shock proteins: a minireview with special emphasis on Csp-family of enteropathogenic *Yersinia*. *Front. Microbiol.* 7:1151. 10.3389/fmicb.2016.01151 27499753 PMC4956666

[B54] Kragh M. L. Muchaamba F. Tasara T. Hansen L. T. (2020). Cold-shock proteins affect desiccation tolerance, biofilm formation and motility in *Listeria monocytogenes*. *Int. J. Food Microbiol.* 329:108662. 10.1016/j.ijfoodmicro.2020.108662 32505890

[B55] Kropac A. C. Eshwar A. K. Stephan R. Tasara T. (2019). New insights on the role of the pLMST6 plasmid in *Listeria monocytogenes* biocide tolerance and virulence. *Front. Microbiol.* 10:1538. 10.3389/fmicb.2019.01538 31338084 PMC6629823

[B56] Lauer P. Chow M. Y. Loessner M. J. Portnoy D. A. Calendar R. (2002). Construction, characterization, and use of two *Listeria monocytogenes* site-specific phage integration vectors. *J. Bacteriol.* 184 4177–4186. 10.1128/jb.184.15.4177-4186.2002 12107135 PMC135211

[B57] Liu Y. Morgan S. Ream A. Huang L. (2013). Gene expression profiling of a nisin-sensitive *Listeria monocytogenes* scott A ctsR deletion mutant. *J. Ind. Microbiol. Biotechnol.* 40 495–505. 10.1007/s10295-013-1243-0 23494707

[B58] Loepfe C. Raimann E. Stephan R. Tasara T. (2010). Reduced host cell invasiveness and oxidative stress tolerance in double and triple csp gene family deletion mutants of *Listeria monocytogenes*. *Foodborne Pathog. Dis.* 7 775–783. 10.1089/fpd.2009.0458 20184451

[B59] Lubelski J. de Jong A. van Merkerk R. Agustiandari H. Kuipers O. P. Kok J. (2006). LmrCD is a major multidrug resistance transporter in *Lactococcus lactis*. *Mol. Microbiol.* 61 771–781. 10.1111/j.1365-2958.2006.05267.x 16879641

[B60] Malekmohammadi S. Kodjovi K. K. Sherwood J. Bergholz T. M. (2017). Genetic and environmental factors influence *Listeria monocytogenes* nisin resistance. *J. Appl. Microbiol.* 123 262–270. 10.1111/jam.13479 28452154

[B61] Mandin P. Fsihi H. Dussurget O. Vergassola M. Milohanic E. Toledo- Arana A. (2005). VirR, a response regulator critical for *Listeria monocytogenes* virulence. *Mol. Microbiol.* 57 1367–1380. 10.1111/j.1365-2958.2005.04776.x 16102006

[B62] Martinez-Rios V. Pedersen M. Pedrazzi M. Gkogka E. Smedsgaard J. Dalgaard P. (2021). Antimicrobial effect of nisin in processed cheese – Quantification of residual nisin by LC-MS/MS and development of new growth and growth boundary model for *Listeria monocytogenes*. *Int. J. Food Microbiol.* 338:108952. 10.1016/j.ijfoodmicro.2020.108952 33229046

[B63] Mascher T. Zimmer S. L. Smith T. A. Helmann J. D. (2004). Antibiotic-inducible promoter regulated by the cell envelope stress-sensing two- component system LiaRS of *Bacillus subtilis*. *Antimicrob. Agents Chemother.* 48 2888–2896. 10.1128/AAC.48.8.2888-2896.2004 15273097 PMC478541

[B64] Michaux C. Holmqvist E. Vasicek E. Sharan M. Barquist L. Westermann A. J. (2017). RNA target profiles direct the discovery of virulence functions for the cold-shock proteins CspC and CspE. *Proc. Natl. Acad. Sci. U.S.A.* 114 6824–6829. 10.1073/pnas.1620772114 28611217 PMC5495234

[B65] Michaux C. Martini C. Shioya K. Lecheheb S. Budin-Verneull A. Cosette P. (2012). CspR, a cold shock RNA-binding protein involved in the long-term survival and the virulence of *Enterococcus faecalis*. *J. Bacteriol.* 194 6900–6908. 10.1128/JB.01673-12 23086208 PMC3510560

[B66] Muchaamba F. Stephan R. Tasara T. (2021). *Listeria monocytogenes* cold shock proteins: small proteins with a huge impact. *Microorganisms* 9:1061. 10.3390/microorganisms9051061 34068949 PMC8155936

[B67] NicAogáin K. O’Byrne C. P. (2016). The role of stress and stress adaptations in determining the fate of the bacterial pathogen listeria monocytogenes in the food chain. *Front. Microbiol.* 7:1865. 10.3389/fmicb.2016.01865 27933042 PMC5120093

[B68] Nielsen P. K. Andersen A. Z. Mols M. van der Veen S. Abee T. Kallipolitis B. H. (2012). Genome-wide transcriptional profiling of the cell envelope stress response and the role of LisRK and CesRK in *Listeria monocytogenes*. *Microbiology* 158 963–974. 10.1099/mic.0.055467-0 22282521

[B69] Palmer M. E. Wiedmann M. Boor K. J. (2009). sigma(B) and sigma(L) contribute to *Listeria monocytogenes* 10403S response to the antimicrobial peptides SdpC and nisin. *Foodborne Pathog. Dis.* 6 1057–1065. 10.1089/fpd.2009.0292 19642919 PMC3145169

[B70] Paul D. (2013). Osmotic stress adaptations in rhizobacteria. *J. Basic Microbiol.* 53 101–110. 10.1002/jobm.201100288 22581676

[B71] Phadtare S. Severinov K. (2010). RNA remodeling and gene regulation by cold shock proteins. *RNA Biol.* 7 788–795. 10.4161/rna.7.6.13482 21045540 PMC3073336

[B72] Phadtare S. Severinov K. (2016). “Gene regulation by cold shock proteins via transcription antitermination,” in *Stress and Environmental Regulation of Gene Expression and Adaptation in Bacteria*, ed. De Bruijn F. (Hoboken, NJ: John Wiley & Sons, Inc), 827–836. 10.1002/9781119004813.ch80

[B73] Phadtare S. Inouye M. Severinov K. (2002). The nucleic acid melting activity of *Escherichia coli* CspE is critical for transcription antitermination and cold acclimation of cells. *J. Biol. Chem.* 277 7239–7245. 10.1074/jbc.M111496200 11756430

[B74] Pinilla C. M. B. Stincone P. Brandelli A. (2021). Proteomic analysis reveals differential responses of *Listeria monocytogenes* to free and nanoencapsulated nisin. *Int. J. Food Microbiol.* 346:109170. 10.1016/j.ijfoodmicro.2021.109170 33770680

[B75] Sahukhal G. S. Elasri M. O. (2014). Identification and characterization of an operon, msaABCR, that controls virulence and biofilm development in *Staphylococcus aureus*. *BMC Microbiol.* 14:154. 10.1186/1471-2180-14-154 24915884 PMC4229872

[B76] Salazar J. K. Wu Z. McMullen P. D. Luo Q. Freitag N. E. Tortorello M. L. (2013). PrfA-like transcription factor gene lmo0753 contributes to L-rhamnose utilization in *Listeria monocytogenes* strains associated with human food-borne infections. *Appl. Environ. Microbiol.* 79 5584–5592. 10.1128/AEM.01812-13 23835178 PMC3754159

[B77] Santos J. C. P. Sousa R. C. S. Otoni C. G. Moraes A. R. F. Souza V. G. L. Medeiros E. A. A. (2018). Nisin and other antimicrobial peptides: production, mechanisms of action, and application in active food packaging. *Innov. Food Sci. Emerg. Technol.* 48 179–194. 10.1016/J.IFSET.2018.06.008

[B78] Schärer K. Stephan R. Tasara T. (2013). Cold shock proteins contribute to the regulation of listeriolysin O production in *Listeria monocytogenes*. *Foodborne Pathog. Dis.* 10 1023–1029. 10.1089/fpd.2013.1562 23952475

[B79] Schmid B. Klumpp J. Raimann E. Loessner M. J. Stephan R. Tasara T. (2009). Role of cold shock proteins in growth of *Listeria monocytogenes* under cold and osmotic stress conditions. *Appl. Environ. Microbiol.* 75 1621–1627. 19151183 10.1128/AEM.02154-08 PMC2655451

[B80] Silhavy T. J. Kahne D. Walker S. (2010). The bacterial cell envelope. *Cold Spring Harb. Perspect. Biol.* 2:a000414. 10.1101/cshperspect.a000414 20452953 PMC2857177

[B81] Stincone P. Miyamoto K. N. Timbe P. P. R. Lieske I. Brandelli A. (2020). Nisin influence on the expression of *Listeria monocytogenes* surface proteins. *J. Proteomics* 226:103906. 10.1016/j.jprot.2020.103906 32707233

[B82] Tasara T. Stephan R. (2007). Evaluation of housekeeping genes in *Listeria monocytogenes* as potential internal control references for normalizing mRNA expression levels in stress adaptation models using real-time PCR. *FEMS Microbiol. Lett.* 269 265–272. 10.1111/j.1574-6968.2007.00633.x 17263845

[B83] Thedieck K. Hain T. Mohamed W. Tindall B. J. Nimtz M. Chakraborty T. (2006). The MprF protein is required for lysinylation of phospholipids in listerial membranes and confers resistance to cationic antimicrobial peptides (CAMPs) on *Listeria monocytogenes*. *Mol. Microbiol.* 62 1325–1339. 10.1111/j.1365-2958.2006.05452.x 17042784

[B84] Velamakanni S. Yao Y. Gutmann D. A. van Veen H. W. (2008). Multidrug transport by the ABC transporter Sav1866 from *Staphylococcus aureus*. *Biochemistry* 47 9300–9308. 10.1021/bi8006737 18690712

[B85] Wambui J. Eshwar A. K. Aalto-Araneda M. Pöntinen A. Stevens M. J. A. Njage P. M. K. (2020). The analysis of field strains isolated from food, animal and clinical sources uncovers natural mutations in *Listeria monocytogenes* nisin resistance genes. *Front. Microbiol.* 11:549531. 10.3389/fmicb.2020.549531 33123101 PMC7574537

[B86] Wang Z. Liu W. Wu T. Bie P. Wu Q. (2016). RNA-seq reveals the critical role of CspA in regulating *Brucella melitensis* metabolism and virulence. *Sci. China Life Sci.* 59 417–424. 10.1007/s11427-015-4981-6 26740105

[B87] Wang Z. Wang S. Wu Q. (2014). Cold shock protein A plays an important role in the stress adaptation and virulence of *Brucella melitensis*. *FEMS Microbiol. Lett.* 354 27–36. 10.1111/1574-6968.12430 24661136

[B88] Watanakunakorn C. (1984). Mode of action and in-vitro activity of vancomycin. *J. Antimicrob. Chemother.* 14(Suppl. D) 7–18. 10.1093/jac/14.suppl_d.7 6440886

[B89] Wiedemann I. Breukink E. van Kraaij C. Kuipers O. P. Bierbaum G. de Kruijff B. (2001). Specific binding of nisin to the peptidoglycan precursor lipid II combines pore formation and inhibition of cell wall biosynthesis for potent antibiotic activity. *J. Biol. Chem.* 276 1772–1779. 10.1074/jbc.M006770200 11038353

[B90] Wiktorczyk-Kapischke N. Skowron K. Grudlewska-Buda K. Wałecka-Zacharska E. Korkus J. Gospodarek-Komkowska E. (2021). Adaptive response of *Listeria monocytogenes* to the stress factors in the food processing environment. *Front. Microbiol.* 12:710085. 10.3389/fmicb.2021.710085 34489900 PMC8417233

[B91] Willimsky G. Bang H. Fischer G. Marahiel M. A. (1992). Characterization of cspB, a *Bacillus subtilis* inducible cold shock gene affecting cell viability at low temperatures. *J. Bacteriol.* 174 6326–6335. 10.1128/jb.174.20.6326-6335.1992 1400185 PMC207576

[B92] Wu R. A. Yuk H. Liu D. Ding T. (2021). Recent advances in understanding the effect of acid-adaptation on the cross-protection to food-related stress of common foodborne pathogens. *Crit. Rev. Food Sci. Nutr.* 27, 1–18. 10.1080/10408398.2021.1913570 33905268

[B93] Wu S. Yu P.-L. Wheeler D. Flint S. (2018). Transcriptomic study on persistence and survival of *Listeria monocytogenes* following lethal treatment with nisin. *J. Glob. Antimicrob. Resist.* 15 25–31. 10.1016/j.jgar.2018.06.003 29933119

[B94] Zhou H. Fang J. Tian Y. Lu X. Y. (2014). Mechanisms of nisin resistance in Gram-positive bacteria. *Ann. Microbiol.* 64 413–420. 10.1007/s13213-013-0679-9

