# Supplementary material for: Cold Shock Proteins Promote Nisin Tolerance in Listeria monocytogenes Through Modulation of Cell Envelope Modification Responses
Source: Front Microbiol. 2021 Dec 24;12:811939. doi: 10.3389/fmicb.2021.811939 (PMC8740179; doi:10.3389/fmicb.2021.811939)
Supplement: Supplementary file 1 [file Table_1.DOCX]

Supplementary Material

**Supplementary Table S1**. RT-qPCR Primers used in this study

| **Primer** | **DNA sequence** | **Gene** | **lmo** | **Reference** |
| --- | --- | --- | --- | --- |
| *cspA* fw | AACATGGAACAAGGTACAG | *cspA* | *lmo1364* | This study |
| *cspA* rv | GTTGGCCTTCTTCAACG |  |  |  |
| *cspB* fw | CAAACAGGTACAGTTAAATGGTTTA | *cspB* | *lmo2016* | This study |
| *cspB* rv | ACGATTTCAAATTCAACGCTTTGA |  |  |  |
| *cspD* fw | TACGGTTTTATCGAATCAGAC | *cspD* | *lmo1879* | This study |
| *cspD* rv | ACGTTAGCTGCTTGAG |  |  |  |
| 16S rRNA | CTTCCGCAATGGACGAAAGT | *16S rRNA* |  | Eshwar et al., 2017 |
| 16S rRNA | CTCATCGTTTACGGCGTG |  |  |  |
| *virR* fw | AACCAGTTGATCTGGATG | *virR* | *lmo1745* | This study |
| *virR* rv | CATCTTCCCAAAGTGCG |  |  |  |
| *dltA* fw | GCCTATCGCACAGGAG | *dltA* | *lmo0974* | This study |
| *dltA* rv | GTTGGGATGACCTGAGC |  |  |  |
| *mprF* fw | GTGGCTAGATGGTAGGG | *mprF* | *lmo1695* | This study |
| *mprF* rv | GTGCCATACCGGCATT |  |  |  |
| *cesR* fw | TAACGGTTGGTGGCAAA | *cesR* | *lmo2422* | This study |
| *cesR* rv | AGCCTCGCGTAACTTAT |  |  |  |
| *lisK* fw | AGTATTCAGGTGACGGC | *lisK* | *lmo1378* | This study |
| *lisK* rv | CAATCGCAAGTCCGAG |  |  |  |
| *liaR* fw | AGAGAACGGTCGTGAAG | *liaR* | *lmo1022* | This study |
| *liaR* rv | CTGGTTTAGCGGTTAGCC |  |  |  |
| *anrB* fw | GCTCACAGTGTCGAAT | *anrB* | *lmo2115* | This study |
| *anrB* rv | TGTCTCTACGTGTAAGACC |  |  |  |
| *telA* fw | TGACTGCGATTCCACTT | *telA* | *lmo1967* | This study |
| *telA* rv | ATGTCCACGATACCTCT |  |  |  |
| *rmlT* fw | GTAAGCGTTGAAACGCA | *rmlT* | *lmo1085* | This study |
| *rmlT* rv | TGTAGCAACCACTGGAC |  |  |  |
| *lmo0441* fw | GCGAGAGGGTTACCAT | *lmo0441* | *lmo0441* | This study |
| lmo0441 rv | CGGACTAGCCTTGGGA |  |  |  |
| *lmo0540* fw | GTG GGC AAC CTT TAG C | *lmo0540* | *lmo0540* | This study |
| *lmo0540* rv | TGT CCC CTG CAC CAT A |  |  |  |
| *lmo1438* fw | TTG CCA ATG GGG GTT C | *lmo1438* | *lmo1438* | This study |
| *lmo1438* rv | GCG GTG CCT GTC TTA C |  |  |  |
| *lmo2039* fw | CTT CCA CAA GCG AAC G | *lmo2039* | *lmo2039* | This study |
| *lmo2039* rv | GCT ACC CGC AGA TAC G |  |  |  |
| lmo2290 fw | AAGGGTCATACGCACT | *lmo2290* | *lmo2290* | This study |
| lmo2290 rv | AGTCACTAGCGGAGGAA |  |  |  |
| *lmo2754* fw | GAT ACA GTC GGA CTC GT | *lmo2754* | *lmo2754* | This study |
| *lmo2754* rv | CGT CGC TGG CAG TTA G |  |  |  |
