# Supplementary material for: Cold Shock Proteins Promote Nisin Tolerance in Listeria monocytogenes Through Modulation of Cell Envelope Modification Responses
Source: Front Microbiol. 2021 Dec 24;12:811939. doi: 10.3389/fmicb.2021.811939 (PMC8740179; doi:10.3389/fmicb.2021.811939)
Supplement: Supplementary file 2 [file Presentation_1.pptx]

## Slide 1
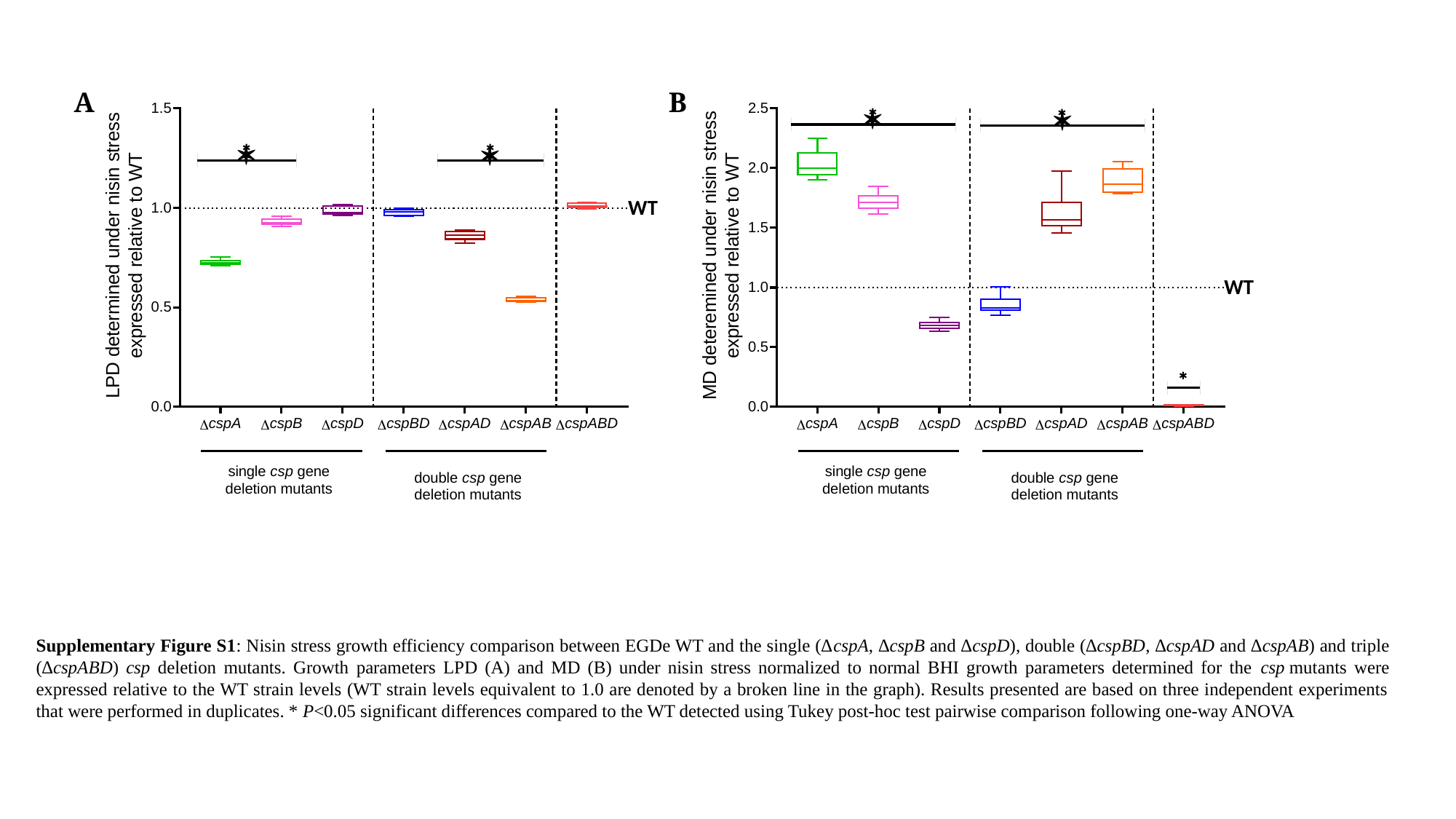

A
B
*
*
*
*
WT
WT
Supplementary Figure S1: Nisin stress growth efficiency comparison between EGDe WT and the single (∆cspA, ∆cspB and ∆cspD), double (∆cspBD, ∆cspAD and ∆cspAB) and triple (∆cspABD) csp deletion mutants. Growth parameters LPD (A) and MD (B) under nisin stress normalized to normal BHI growth parameters determined for the csp mutants were expressed relative to the WT strain levels (WT strain levels equivalent to 1.0 are denoted by a broken line in the graph). Results presented are based on three independent experiments that were performed in duplicates. * P<0.05 significant differences compared to the WT detected using Tukey post-hoc test pairwise comparison following one-way ANOVA

## Slide 2
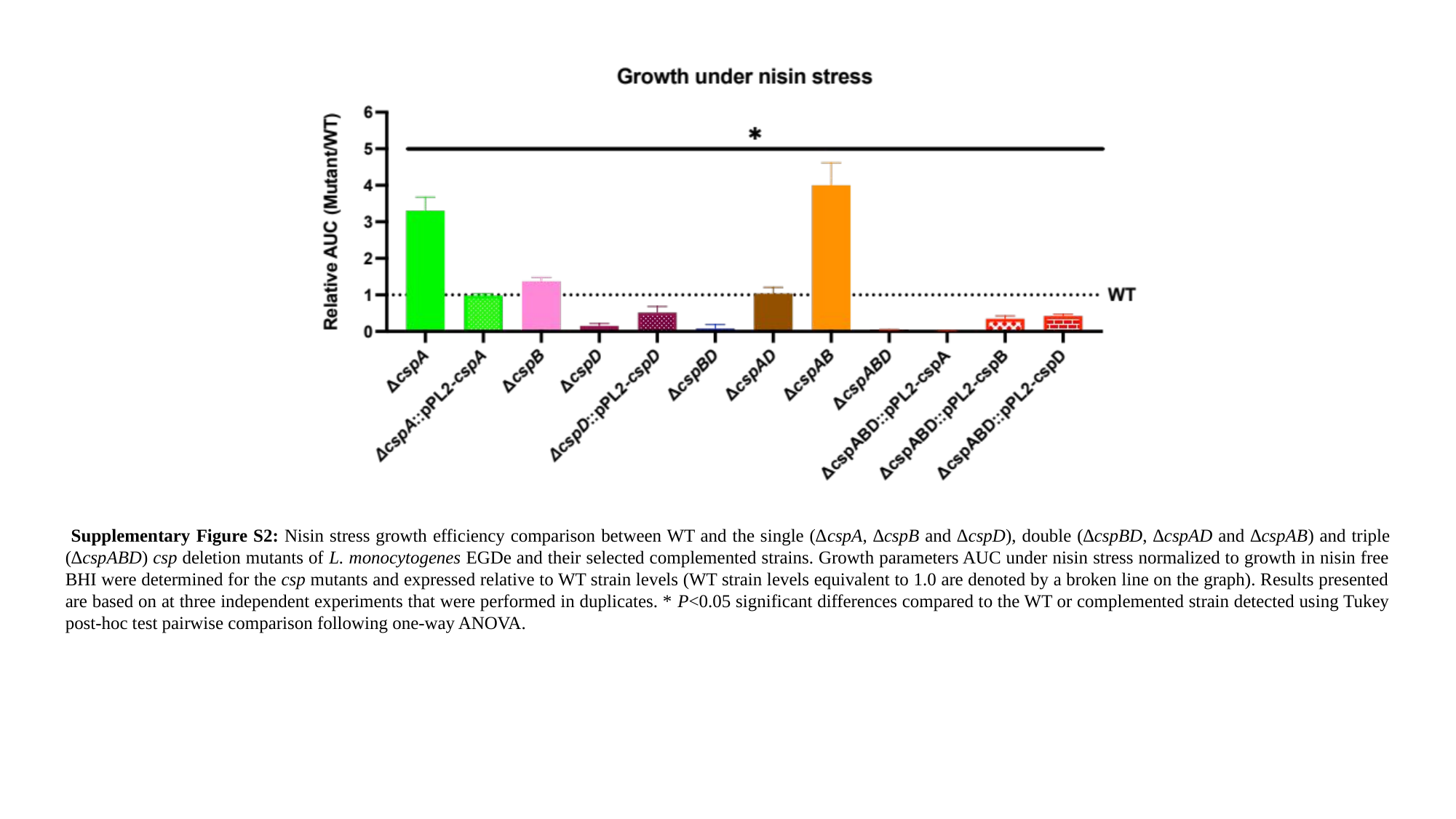

Supplementary Figure S2: Nisin stress growth efficiency comparison between WT and the single (∆cspA, ∆cspB and ∆cspD), double (∆cspBD, ∆cspAD and ∆cspAB) and triple (∆cspABD) csp deletion mutants of L. monocytogenes EGDe and their selected complemented strains. Growth parameters AUC under nisin stress normalized to growth in nisin free BHI were determined for the csp mutants and expressed relative to WT strain levels (WT strain levels equivalent to 1.0 are denoted by a broken line on the graph). Results presented are based on at three independent experiments that were performed in duplicates. * P<0.05 significant differences compared to the WT or complemented strain detected using Tukey post-hoc test pairwise comparison following one-way ANOVA.

## Slide 3
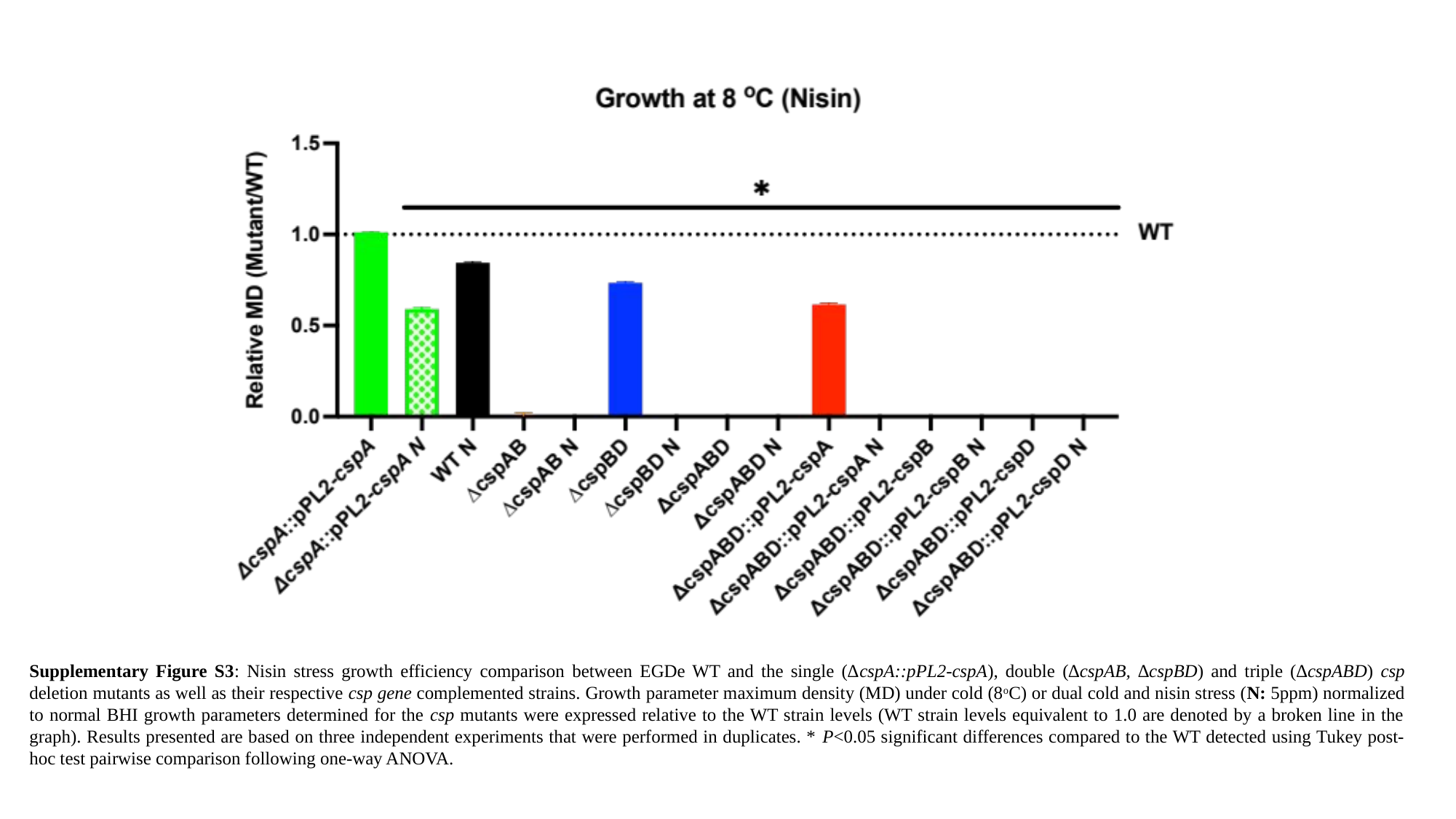

Supplementary Figure S3: Nisin stress growth efficiency comparison between EGDe WT and the single (∆cspA::pPL2-cspA), double (∆cspAB, ∆cspBD) and triple (∆cspABD) csp deletion mutants as well as their respective csp gene complemented strains. Growth parameter maximum density (MD) under cold (8oC) or dual cold and nisin stress (N: 5ppm) normalized to normal BHI growth parameters determined for the csp mutants were expressed relative to the WT strain levels (WT strain levels equivalent to 1.0 are denoted by a broken line in the graph). Results presented are based on three independent experiments that were performed in duplicates. * P<0.05 significant differences compared to the WT detected using Tukey post-hoc test pairwise comparison following one-way ANOVA.

## Slide 4
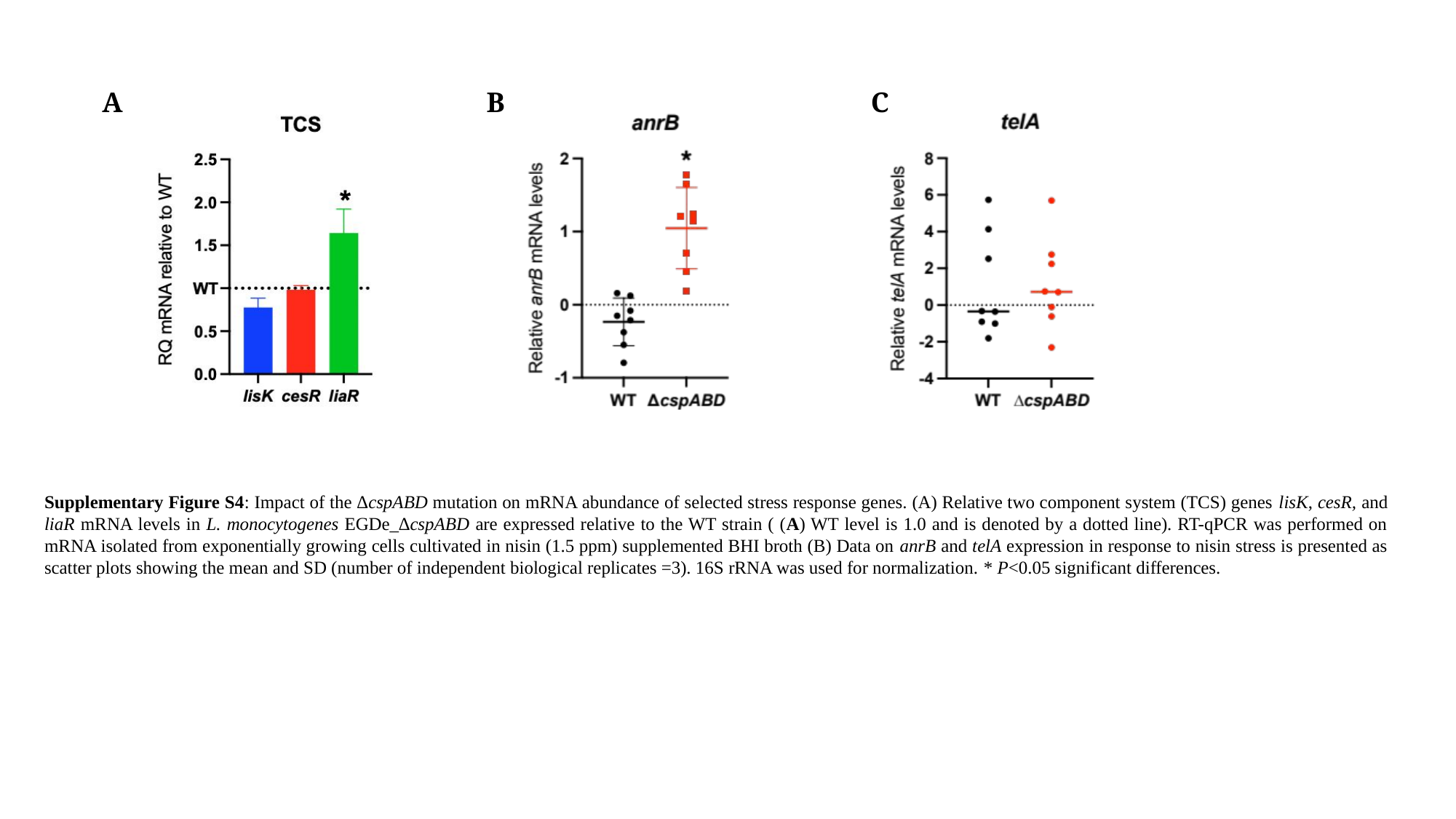

A
B
C
Supplementary Figure S4: Impact of the ∆cspABD mutation on mRNA abundance of selected stress response genes. (A) Relative two component system (TCS) genes lisK, cesR, and liaR mRNA levels in L. monocytogenes EGDe_∆cspABD are expressed relative to the WT strain ( (A) WT level is 1.0 and is denoted by a dotted line). RT-qPCR was performed on mRNA isolated from exponentially growing cells cultivated in nisin (1.5 ppm) supplemented BHI broth (B) Data on anrB and telA expression in response to nisin stress is presented as scatter plots showing the mean and SD (number of independent biological replicates =3). 16S rRNA was used for normalization. * P<0.05 significant differences.

## Slide 5
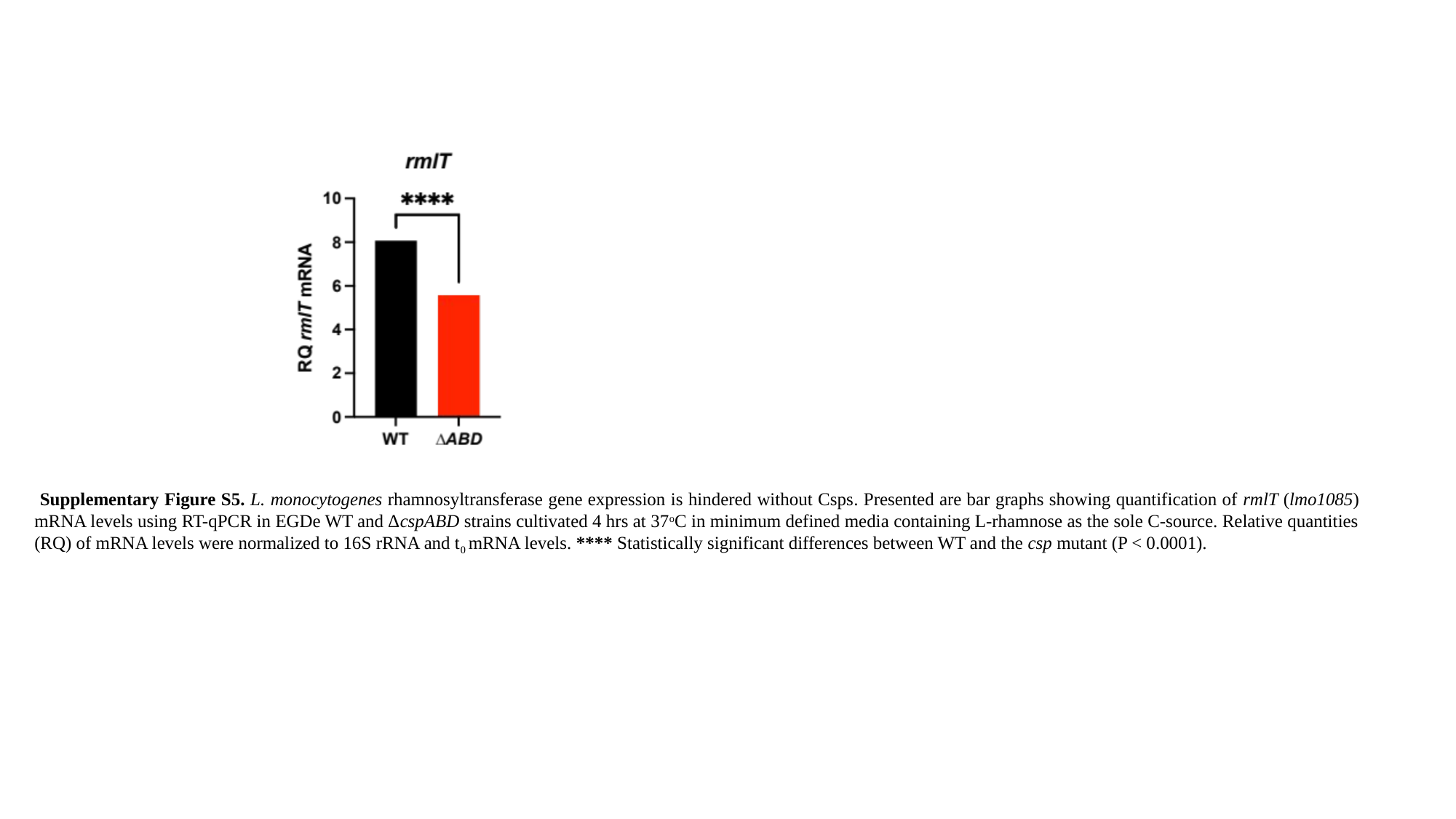

Supplementary Figure S5. L. monocytogenes rhamnosyltransferase gene expression is hindered without Csps. Presented are bar graphs showing quantification of rmlT (lmo1085) mRNA levels using RT-qPCR in EGDe WT and ∆cspABD strains cultivated 4 hrs at 37oC in minimum defined media containing L-rhamnose as the sole C-source. Relative quantities (RQ) of mRNA levels were normalized to 16S rRNA and t0 mRNA levels. **** Statistically significant differences between WT and the csp mutant (P < 0.0001).
